# Video Presentations

**DOI:** 10.1155/1996/82301

**Published:** 1996


### VIDEO PRESENTATIONS

### Topic: LIVER

### V001

### LEFT HEPATECTOMY WITHOUT VASCULAR OCCLUSION FOR LIVER RELATED DONOR

J. Belghiti, R. Noun, D. Jan*, Y. Révilon*

Departments of Digestive Surgery & * Pediatric, Hôpital Beaujon, Clichy, * Hôpital Necker Enfants Malades, Paris, France.

A 30 year old man underwent left lateral segmentectomy for graft-harvesting operation for transplantation of his 3 years daughter with biliary atresia.
Donor liver volumetry using CT scan shows a left lobe of 300 cc. Anatomic analysis of the arterial supply demonstrated a common branche of segments II, III and IV. Anatomic analysis of hepatic veins was obtained by ultrasonography.
Donor operation: Intraoperative US confirmed the presence of a common trunk of the middle and left hepatic veins. The left hepatic artery was exposed near its origin from the proper hepatic artery and the left portal vein was exposed posteriorly near the caudate lobe. The triangular and hepatogastric ligaments were dissected from the liver and the common trunk of the middle and left hepatic veins was isolated.The hepatic parenchyma was transected using ultrasonic aspirator without blood vessel clamping. After parenchyma transection from the right lobe and from the anterior part of the caudate lobe, the left bile duct was transected. When the liver was free on its hepatic artery, portal vein and hepatic vein which were then clamped, the liver graft was flushed in situ through the left portal vein first with 200 mL of Ringer's solution. On the back table the liver was perfused with 1000 mL UW solution and hepatic veins of the graft were prepared. Fibrin glue was sprayed on the cut surface of the liver for secure hemostasis. Blood loss was estimated to 800 mL for wich autotransfusion was performed.
Postoperative course of both donor and recipient were unventefull and they were discharged from the hospital respectively on the 7 and 21 postoperative days.

Lenght of video : 9 minutes

### V002

### CAUDATE LOBE SEGMENTECTOMY.

Calzoni D, Samori G, Ferrandi A, Beati C, Tozzi P.
Surgical Department "M.Donati" - S.Carlo Hospital - Milan - Italy

Segment I resection for colo-rectal carcinoma metastasis is seldom reported in the literature.
The resection can be performed when the lesion is solitary and not adjacent to left portal branch. Neoplastic lesions can be classified into 5 types according to topographic localization (Hasegawa 1991).
The case of a 55 year old woman with metachronous caudate lobe metastasis from sigma adenocarcinoma resected 2 years before is reported.
The caudate lobe lesion was type II: near the inferior side of the caudate.
The lesion was demonstated by preoperative US and porto-CT scan.
The liver was ulteriorly explored by intraoperative US.
The access to the tumour was through the lesser sac after mobilization of the left hepatic lobe.
Division of the hepatic veins on the left side of the inferior vena cava was achieved.
Dissection of the caudate lobe from portal and hepatic branches was made. The segment I was then extirped.
Hepatic I segmentectomy for colon adenocarcinoma metastasis can be easily performed in selected patients.

### V003

### CENTRAL HEPATECTOMY : ANATOMIC RESECTION OF SEGMENTS 4, 5 AND 8

D. Cherqui, B. Tantawi, P.L. Fagniez. Department of Surgery, Université Paris XII - Hôpital Henri Mondor Créteil France

Central liver tumors involving segments 4, 5 and 8 may be removed by right hepatectomy extended to segment 4 (resection of segments 4-8). However, such a resection may be too extensive when the left lobe (segments 2 and 3) is small and may lead to postoperative liver failure. We present an alternative technique in such patients consisting in anatomic resection of segments 4, 5 and 8 that we called central hepatectomy which removes the territory of the middle hepatic vein while preserving the left and right hepatic veins.
The surgical technique includes:

- complete liver mobilization and control of the 3 major hepatic veins
- division of the pedicles of segment 4 on the right border of the round ligament
- opening of the hilar plate and control and division of the right anterior portal pedicle for segments 5 and 8
- liver transsection along anatomic planes defined by ischemic margins
- liver transsection is performed without vascular clamping. If hemorrhage occurs, the portal triad is clamped with or without clamping of the major hepatic veins
- care is taken to preserve the right hepatic vein.

In this video we present the case of a 60 year-old woman with a tumor located at the junction of segments 8 and 4, diagnosed 10 years after treatment for a T2 N0 M0 breast carcinoma. The patient underwent central hepatectomy with an uneventful outcome. Histologic examination showed a metastasis of breast carcinoma.
This technique was used in 4 patients including the case shown here. Indications were secondary liver cancer in 3 cases (colorectal 2, breast 1) and hepatocellular adenoma in 1 case. The patients did not receive any blood tranfusions, vascular clamping was required in 3 cases for 15, 18 and 20 minutes respectively and postoperative course was uneventful.
The technique presented here is a safe alternative to extended right hepatectomy for resection of central liver tumors in patients with a small left lobe. Initial vascular control permits to perform a bloodless and strictly anatomic resection of segments 4, 5 and 8.

### V004

### CAUDATE LOBE AND LEFT HEPATECTOMY EXTENDED TO THE 5TH AND 8TH SEGMENTS FOR HCC IN NORMAL LIVER.

A.Corsini, F.Galeotti, A.M.Ronconi, F.Zingales, B.Rashidi
Divisione Chirurgica II^a, Azienda Ospedaliera di Padova, Padova, Italy.

The case subject of this videotape is a man 59 years old with a large tumor of the caudate lobe involving the 4^th and 8^th segment. The patient, accepted in our Departement after an explorative laparotomy that confirmed the diagnosis of hepatocellular carcinoma in normal liver, underwent a new operation, realizing an en bloc caudate lobe and left hepatectomy extended to the 5^th and 8^th segments, that is a left and medial hepatic vein lobectomy. The remaining liver ( 6^th and 7^th segments) was supplied by the lateral (posterior) branch of the right portal pedicle and drained by the right hepatic vein. Moreover the tumor was infiltrating the anterior wall of the retrohepatic vena cava and thus the hepatectomy was associated with the remove of a little patch of this vein. Parenchimal hemostasis was obtained by early hilar dissection and control of the arterial and portal branches. This procedure was integrated in case of necessity by a Pringle manoeuvre with alternative intervals of 15 minutes of hepatic anoxia and 5 minutes of vascular release and hemostasis of bloody surfaces by pad compression. The suture of the hepatic veins was realized by stapling techniques. The postoperative course was uneventful and the patient is still living and disease free two years after the operation. This case therefore incite to a more aggressive surgical treatement of hepatocellular carcinoma. Lenght of the video 15 minutes.

### V005

### CLOSED TOTAL CYSTOPERICYSTECTOMY FOR LIVER HYDATID CYST ADHERENT TO VENA CAVA AND MAIN HEPATIC VEINS

F. Crucitti, GB. Doglietto, S. Alfieri, F. Pacelli
Department of Surgery, Catholic University, Rome, Italy.

The video shows the surgical approach, performing a closed total cystopericystectomy, for a 8 cm liver hydatid cyst located between V-VIII segment adherent to vena cava and to main hepatic veins.

After an adequate control of subdiaphragmatic and subhepatic inferior vena cava, the operation consists of removing the whole cyst, including the pericystium, in the plane between the pericystium and liver parenchyma, keeping close to the pericyst, a relatively bloodless plane, and just outside the adjacent normal, friable and vascular liver tissue. This plane can be developed quite easily and all vascular and biliary channels are tied as they are encountered during the dissection.

A small portion of pericyst adherent to inferior vena cava and hepatic veins was left in place (subtotal cystopericystectomy). This avoids the risk of massive haemorrhage, a hazard sometimes fatal and not justifiable for a benign lesion.

No postoperative complications occurred. The patient was discharge from the hospital on the VIII post-operative-day and lives for one year without recurrence.

This technique allows radical surgical treatment also for deep cysts of the liver, adherent to vena cava or hepatic veins, avoiding the possibility of recurrence.

### V006

### LIVING RELATED DONOR IN LIVER TRANSPLANTATION

*E. de Santibañes, M. Ciardullo, J. Sivori, J. Pekolj, J. Mattera, J. Grondona.*
General Surgical Service. Liver Transplantation Unit. Hospital Italiano de Buenos Aires. (Argentina)

The lack of cadaveric organ donors for transplants is a worldwide problem.The situation is even worse when dealing with pediatric patients which results in a high mortality rate for those low weight patients awaiting transplantation. During the last decade, new techniques in organ resection such as liver reduction for cadaveric or living related donors (LRD), has allowed us to implement a number of programs which improve the survival rate of those on the waiting lists.The purpose of this paper is to convey our experience at the Hospital Italiano de Buenos Aires with LRD and its impact on the Pediatric Liver Transplant Program. Between January 1988 and March 1994 we have had a total of 83 patients on the waiting list; 45 patients were admitted during the period Jan/88-April 92 (period I) and 38 between March92 and May94 (period II).We performed 42 transplants in 39 patients,20 of these were transplanted during period 1 and 19 during period II. The average age of the 39 patients was 6.3 y.o. (range 0.9-16), 20 females and 19 males. Cadaveric donors were used for 35 transplants:19 full size and 16 reduced livers.In 10 cases during period II we performed the liver transplantation with LRD.Eleven children have received a liver transplant from a living related donor.This procedure has resulted in a 50% decrease in the mortality rate while on the waiting list, 100% survival of the receptors and donors with only minor complications to these.Living related donor liver transplantation should improve the possibilities for small receptors with extrahepatic biliary atresia. In this tape we described the differents steps of the living related liver transplantation:donor hepatectomy, procurement of internal jugular and saphenous vein, back table, recipient hepatectomy and the implant of the new liver in the recipient with the different vascular anastomosis and biliary reconstruction. (The tape lasts 15 minutes)

### V007

### LIGATURE OF VENA CAVA FOR COMPRESSIVE THROMBOSIS BY HYDATIQUE LIVER CYST

G. De Sena - G. Amatucci - F. La Rocca - F. Chianese - P. Festa

Dept. of Surgery - "San G. Moscati" Hospital - Avellino

It is an operative technique who show the ligature of the inferior vena cava for thrombosis by compressive hydatique cyst of right liver.

There was an hemodynamics compensation assured by a new formed collateral circulation.

The vena cava was dissected under confluence of renal veins. During the dissection under diaphragm, the vena cava was injured and fastly repaired, as showed. The ligature was performed upper thrombosis with very-slow adsorbable monifilament suture. The vein wasn't sectionned. It was impossible using the "cava-clip".

The hidatique cyst was treated with any cystopericystectomy.

The ligature of vena cava may be do if there is a good hemodynamic compensation.

This operative technique is right for a risk of an embolism in a patient with thrombosis secundary to the other disease.

### V008

### HETEROTOPIC PARTIAL LIVER ISOTRANSPLANT IN THE RAT

H.J. Duran, L. Lorente, J. Rodriguez, M.C. Duran, G. Rodriguez-Fabian, M.A. Aller, J. Arias.
Department of Surgery. University Hospital "San Carlos". UCM. Madrid. Spain.

Heterotopic or auxiliary liver transplantation is an attractive alternative to the orthotopic liver transplantation and provide temporary support of a potentially reversible liver damage. We have developed an experimental model of heterotopic partial liver isotransplant in the rat using a microsurgical technique so as to study the inter-liver competition.

The auxiliary liver graft consists of the right lateral and caudate lobes. The graft liver was vascularized with blood from the portal vein and the liver of the recipient was only vascularized arterially. The cuff technique for the portal vein anastomosis simplifies the microvascular anastomosis. The venous drainage by the infrahepatic inferior vena cava prevented the graft congestion. The bile duct was passed into the duodenum of the recipient and fixed to the wall by a simple stich.

The graft underwent regeneration during the first 30 days of the postoperative period, it may be considered that the partial heterotopic transplant of the liver supports the hepatic function.

### V009

**MICROSURGICAL CHOLESTASIS IN THE RAT: A NEW EXPERIMENTAL MODEL WHICH AVOIDS BILIARY RECANALIZATION**

H.J. Duran, G. Rodriguez, S. Alonso, M.C. Duran, L. Lorente, M.A. Aller, J. Arias.
Department of Surgery. University Hospital "San Carlos". UCM. Madrid. Spain

Extrahepatic cholestasis in the rat based on the section of the bile duct between ligatures causes a high incidence of mortality and sepsis with multiple abscesses in the intraperitoneal, hepatic and pulmonary areas.

An experimental model of extrahepatic cholestasis in Wistar rats, using a microsurgical technique, was performed. The bile ducts that drain the four hepatic lobes in continuity with the bile duct were resected and the postoperative evolution at 7th (n=10), 16th (n=10), 30th (n=9) and 37th (n=7) days was studied.

All of the animals were alive and had jaundice, body weight loss statistically significant, hepatomegaly, splenomegaly, hepatic and pulmonar samplex in the animals in regards to the control rats. Serum concentration of bilirubin ($p<0.001$), bile acids ($p<0.001$), alkaline phosphatase ($p<0.05$) and gamma-glutamyltranspeptidase ($p<0.01$) increased in relation to those of the control group.
Left splenorrenal (16 days p.o.) and paraesophageal (30 and 37 days p.o.) collateral circulation was observed. Intense biliary proliferation in the portal spaces and in areas 1 (16 days p.o.), 2 (30 days p.o.) and 3 of the acini with hepatocytic necrosis (37 days p.o.) were observed.

The use of this technique prevents the development of hepatic cysts and hepatoneumonic abscesses, complications inherent in the bile-duct ligation techniques, as well as the biliary recanalization in the postoperative period.

### V010

LAPAROSCOPIC RESECTION OF LIVER ( SEGMENT 3 ) HAEMANGIOMA.

M.Filauro,C. Bagarolo, E. Ciferri, G.M. Gazzaniga
1st Surgical Dep., S.Martino General Hospital, Genoa, Italy

Focal lesions of the liver are rarely treated using laparoscopic approach.
Authors presents the case of a 45 years-old female , admitted inSurgical Departement with upper abdominal pain , mainly left sided nausea , vomiting , slight fever .
Haematological checks revealed only WBC increase and slight elevation of transaminases values .
Ultrasuond examination of the abdomen showed gallbladder stones with moderate signs of cholecistitis , and a large lesion of inferior lateral segment of left lobe of the liver (5 x 4 cm.)
Angio-CT scan of upper abdomen confirmed the hepatic mass , with typical contrast enhancement of hepatic haemangioma.
Laparoscopic approach was prevented , with the aim of left inferior lateral segmentectomy , and cholecistectomy.
Trocar placement was scheduled to obtain a good vision of the all liver and to be able to insert different laparoscopic tools. Pringle manoeuvre was realized by a soft bowel forceps.
Liver section was obtained using monopolar forceps , metallic clips , and major vascular pedicles were severed using 30 mm reloadble EndoGIA.
Cholecistectomy was also performed , and a silastic drainage positioned near the hepatic surface.
Postoperative course was uneventful , and patient was discharged after 8 days from operation.
Follow-up revealed an optimal hepatic growth , with complete parenchimal regeneration at 6 month from surgical approach.
Laparoscopic liver resections are technically feasible in selected cases and by the use of appropriate endosurgical instruments.

### V011

**SHUNT THERAPY FOR BUDD-CHIARI SYNDROME (10')**

*E.Forni, F.Meriggi, G.Morone*
University of Pavia (Italy), General Surgical Clinic, IRCCS SanMatteo Hospital, Hepato-biliary Surgical Unit.

Surgical treatment of Budd-Chiari syndrome consists in porto-systemic decompression or in liver transplantation.
This video shows the operation on a 28-year-old man with the complete thrombosis of the hepatic veins and the incomplete thrombosis of the suprahepatic inferior vena cava, caused by a myeloproliferative disease. Hepatomegaly, refractory ascites and liver functional failure were present. There was not encephalopahty. After doppler ultrasonography, angiography, CT scan and MRI, we decided to perform a double prosthetic side to side portal-caval and caval-atrial bypass to decompress the hypertensive IVC and to shunt the entire portal venous flow.
Through a bilateral subcostal laparotomy the inferior vena cava and the portal vein are dissected and isolated. The side to side portal-caval (suprarenal) bypass with a 10 mm ring reinforced PTFE graft is performed. Then, after a median stemotomy, the lateral wall of the right atrium is exposed and a side to side caval (infrarenal)-atrial shunt is achieved with a 16 mm ring reinforced graft. The graft is 50 cm long and passes anteriorly to the stomach and to the left lobe of the liver into the mediastinum through a hole made in the anterior diaphragm.
Pre-shunt pressure: IVC 22 cm $H_2O$, portal vein 33 cm $H_2O$.
Post-shunt pressure: IVC 5 cm $H_2O$, portal vein 15 cm $H_2O$.
There were no postoperative complications; the ascites disappeared, the liver function improved and there were no signs of encephalopathy. After 2 years, doppler ultrasonography confirms a good flow in both grafts.

### V012

HETEROTOPIC LIVER TRANSPLANTATION IN THE LEFT HYPOCHONDRIUM: THE FUTURE OF THE NATIVE LIVER

G. Fourtanier, B. Suc, J.L. Rumeau, D. Durand

This video concerns the long term follow up of the native liver after heterotopic liver transplantation (HLT). The patient was operated in 1989 for autoimmune cirrhosis. Because of a thrombosis of the portal vein found peroperatively, an HLT ws done in the left hypochondrium after splenectomy. The immediate follow up was uneventful and isotopic scintigraphies show the functional graft and the progressive atrophy of the native liver. Five years after, an hepatocarcinoma on the native liver is found after increasing of Alpha Foeto Protein and Lipiodol angiography. A total hepatectomy is performed and the patient is quite well 18 months later.

### V013

### MAJOR LIVER RESECTION ON ABNORMAL LIVER : PROTECTION WITH HYPOTHERMIC PERFUSION IN SITU

L. Hannoun, C. Penna
Department of Surgery, St-Antoine Hospital, Paris, France

Total vascular exclusion of the liver allows major liver resection for hypervascularized tumors involving main portal or hepatic vein pedicles. However, liver ischemia is not well tolerated by cirrhotic, cholestatic or fibrotic livers. We have previously showed that these abnormal livers could be protected using an in situ perfusion of UW, in the same way than normal liver grafts during liver transplantation.

**Presentation of the case**
A 50-year-old man presented with an hepatocellular carcinoma developed on a fibrotic liver (hepatitis B and C). The lesion was 15 cm in diameter and was developed in the right lobe of the liver and the segment 4. The portal confluence was laminated but the portal vein was not invaded. Posteriorly, the tumor laminated the right and the median hepatic veins and was in contact with the anterior side of the vena cava.
**Surgical procedure**
A large bi subcostal incision was performed. The liver was fully mobilized by section of it's ligaments and the entire vena cava was liberated from the posterior face of the liver. A cannula was inserted in the right hepatic artery and was directed towards the left hepatic artery. A total vascular occlusion was obtained by clamping the portal pedicle and the supra and infra hepatic segments of the vena cava. The liver was then perfused with UW at 4° C and the effluent was drained via a small cavotomy. An extented right lobectomy was then performed with a section of the main vascular pedicles using a TA stapler. The cannula was then removed, the cavotomy sutured and the vascular clamps removed. The hemostasis was satisfactory and the patients did not received blood transfusion. The ischemia lasted for 90 min.
**Results**
The post operative course was uneventful. The patient did not developed jaundice or encephalopathy. At postoperative day 2 the prothrombine time was at 56 %.
**Conclusion**
This case and others from our institution are encouraging, however the principle of protection of abnormal livers by perfusion in situ with UW remains to be demonstrated.

### V014

### LAPAROSCOPIC FENESTRATION OF POLYCYCTIC LIVER DISEASE

W. John B. Hodgson
Department of Surgery, The Brooklyn Hospital Center, Brooklyn, N.Y.

Single liver cysts have been treated by laparoscopic drainage, decompression and excision since 1991.
Multiple congenital cysts of the liver (polycystic disease) represent a quite different challenge. There are so many that they can cause severe abdominal distension and the pressure effect on liver parenchyma may cause hepatic failure. Open partial hepatic resection and fenestration of the anterior cysts have been done to control this disease but is tedious and difficult, and may be associated with high volume fluid loss.
A laparoscopic hepatic multi cystic decompression is presented in a 44 year old lady had over twenty cysts excised, including deep cysts, into which the camera could be placed to determine whether or not further dissection should be done. Thin areas of cysts were excised by Endoshears and areas with parenchymal involvement were divided with an Endo GIA. No drains were used.
Postoperatively the patient had a transient rise in bilirubin but was able to be discharged in three days. Three years later she was symptom free.
It is not advised that this procedure is carried out unless the surgeon is already a hepatobiliary specialist, as a knowledge of hepatic anatomy is essential.
The video will be edited to 8 minutes.

### V015

### THE SCALPEL TECHNIQUE OF LIVER RESECTION

A.M. Isla, H. Sawada, J.B. Perez, G. Zografos, L. Grego, N. DeRuvo, N.A. Habib.

**One of the most frequent complications of liver surgery is intraoperative bleeding. Postoperative mortality has been related to multiple transfusions so, all measures that permit control of bleeding would eventually, reduce the overall mortality and morbidity.**

**In the video we present "The scalpel technique for liver resection" that consists of total vascular exclusion (TVE) of the liver and bloodless transection of the parenchyma with a knife, and oversewing of the venous, arterial, portal, and biliary branches. We show the necessary steps for the TVE as well as for the scalpel transection and haemostasis.**

**This technique has been used successfully in non-cirrhotic, cirrhotic and obstructed livers.**

### V016

### THE NEW APPROACH OF TRANSTHORACIC ESOPHAGEAL TRANSECTION (THE SUGIURA-FUTAGAWA PROCEDURE)

K. Kojima, K. Oohashi, S. Ooura, R. Nakanishi, M. Fukasawa, T. Beppu, S. Futagawa
2nd Department of Surgery, Juntendo University, Tokyo, Japan

The transthoracic esophageal transection (Sugiura-Futagawa Procedure) for esophageal varices consists of transthoracic esophageal transection and devascularization, and transabdominal esophagogastric devascularization including splenectomy and pyloroplasty. This procedure have been made for poor risk cases in 2-staged operation, transthoracic and transabdominal processes, in order to lessen the surgical stress and to assure the safety, but since 1990s, it has been performed in the one-staged transthoracic esophageal transection by the thoraco-abdominal approach through the 1t. 7-th intercostal space.
Thirty-one cases have been operated by this method by May, 1995. They were 15 males and 16 females, 20 liver cirrhosis, 7 idiopathic portal hypertension, 3 extra-hepatic portal vein occlusion and 1 other. The average operation time of this method was 327min (211-590min), the average intraoperative bleeding volume was 817ml(200-2650ml), and the number of cases completed operation without blood transfusion was 17 (55%). The number of cases of sutural insufficiency at anastomotic site including minor leakage was 4 (13%). There was no experience of death during hospitalization. The indications of this method are Child A and B cases, showing total bilirubin $\leq$2.5mg/dl, albumin $\geq$3.5g/dl and prothrombin activity $\geq$50%.
The advantage of this method are 1) unnecessary of intraoperative postural change, 2) possible to keep a favorable visual field, and 3) unnecessary of costectomy at posterior area of thoracotomied wound and alleviation of postoperative pain.
The case shown in this video is an esophagogastric varices in a female of 52 years old with liver cirrhosis, Child A and R15-ICG 22%, and history of hematoemesis(+). The operation time was 211 min, the bleeding volume was 200ml and the weight of the excised spleen was 415g.
The length of the video is about 15 min.

### V017

### LIVER RESECTION: IMPROVED SEGMENTAL RESECTION OF THE RIGHT LIVER - THE POSTERIOR INTRAHEPATIC GLISSONIAN APPROACH

B. Launois, G.G. Jamieson, J. Terblanche
Department of Digestive Surgery and Transplant Unit, CHR Pontchaillou, Rue Henri Le Guilloux, 35033 Rennes, France

A new technique, which has simplified segmental liver resection, is described. The individual Glissonian sheaths supplying the segments of the right liver are approached by posterior intrahepatic dissection from the porta hepatis. The segment to be removed is clearly delineated by clamping individual sheaths which produces a colour change. This permits accurate resection of a single liver segment.

### V018

### LIVING-RELATED LIVER TRANSPLANTATION USING SEGMENT 2,3 and 4

SG LEE, KM KIM*, YJ LEE, KM PARK

Department of Surgery, Pediatric*, Asan MEDICAL Center, University of Ulsan, Seoul, Korea

A 15-month-old boy with manifestations of jaundice and marked splenomegaly underwent LRLT on July 20th,1995. After birth, progressive jaundice developed, and he underwent exploration 2 months later and was finally diagnosed as intrahepatic bile duct paucity. At the time of LRLT, his weight was 9.1kg and CT revealed seperate drainage of V2 and V3 into IVC and MHV respectively. His mother, aged 25 years, volunteered to become a living donor. She weighed 45.4kg and her left lobe(segment 2,3,4) was estimated 190 gm by CT volumetry. Because donor's left lobe was exceptionally small and variant of recipient's hepatic vein drainage, we decided to use donor's left lobe instead of lateral segment as graft for convenience of HV anastomosis. However, actual weight of left lobe graft was 260gm and it's size was too big to close abdomen primarily. After primary closure of abdominal wall by force, serum transaminase was raised over 2,500 IU/L immediately after operation. On the next day, serum transaminase fall below 1,000 IU/L and thereafter, recovery was very smooth. This video shows all procedures of LRLT using segment 2,3 and 4 in both donor and recipient.

### V019

### RIGHT ANTERIOR SEGMENTECTOMY

M. Makuuchi, S. Kawasaki, S, Miyagawa, T. Kakazu
Second Department of Surery, University of Tokyo, Tokyo, Japan
First Department of Surgery, Shinshu University, Matsumoto, Japan

This film presents surgical techniques of right anterior segmentectomy.
The patient is a 56-year-old man with metastatic liver cancer from the colon. Sigmoidectomy was performed on October 1991 in the other hospital. Liver metastasis was found by US and he was referred to our hospital. Two tumors in the segment VIII and V were found and the latter one infiltrated the right anterior portal pedicle. CT volumetry disclosed that the left liver volume was 30 % of the whole liver, so that right hemihepatectomy was abandoned and right anteior segmentectomy right paramedian sectoriectomy was selected.
With J-shaped incision extended to the ninth intercostal space, laparotomy was accomplished. After cholecystectomy, the right anterior bile duct, hepatic artery and portal vein were ligated and divided. Other portal venous and hepatic arterial branches were taped. The discolored area coresponding to the right anterior segment was marked with electrocautery. Under hemihepatic occlusion of the left liver, division of the liver parenchya was undergone along the major fissure. The middle hepatic vein was completely exposed. Then, selective vascular occlusion was switched to the right posterior segment and liver transection was carried out along the intersegmental plane between the right anterior and posterior segments. After about the caudal half of this plane was divided, the right anterior portal pedicle was ligated and severed. Liver transection was then proceeded along the right hepatic vein and it was completely exposed.
By this procedure, the right anterior segment was resected anatomically. On the raw surface of the liver, the middle and right heapatic veins could be seen from the confluence to the inferior vena cava to the peripheral small tributaries of them.

### V020

### AN OPTIMUM SURGICAL TECHNIQUE OF LIVER HYDATIC CYST TO AVOID SPILLAGE OF HYDATIC CYST

S. Mallagray, T. Butrón, MJ. Castillo, A. Garcia.

Department of Surgery. Cruz RoJa Hospital and Doce de Octubre Hospital, Madrid, Spain.

One of the problems in the surgical treatment of huge hydatic cysts is the possiibility of spillage of hydatic membranes and liquid in the abdominal cavity. We propose a close total pericystectomy or an open total pericystectomy using a trocar attached to a negative pressure suction apparatus to avoid this problem.
MATERIAL AND METHODS. Supported by the results of a prospective study of 48 patients operated on with one or more huge hydatic cysts, at least one located in posterosuperior segments of the liver. We show two cases of hepatic hydatic cyst treated surgically with a close total pericystectomy and an open total pericystectomy, respectively, by a thoracophrenolaparotomy by superior edge of ten rib (TPL10).
RESULTS. With TPL10 we had a large operative field performing radical techniques in all patients. We used a modified trocar of Dermileau attached to a negative pressure suction apparatus that allowed a sudden aspiration of the cyst in the open pericystectomy. After we performed total pericystectomy and commun duct exploration if there was biliary fistula. There were not recidivation in any patients.
CONCLUSIONS. TPL10 allowed perform radical techniques in almost all cases. Aclose total pericystectomy and the use of atrocar attached to a negative pressure suction apparatus in the open total pericystectomy avoided spillage of hydatic cyst.

### V021

### LIVER TRANSPLANTATION WITH PRESERVATION OF RETROHEPATIC RECIPIENT VENA CAVA

Mazziotti A, Jovine E, Grazi GL, Masetti M, Pierangeli F, Morganti M, and Cavallari A.
2nd Dept Surgery, University of Bologna, S.Orsola Hospital, Via Massarenti 9, 40138 Bologna, Italy

The video shows the technique of orthotopic liver transplantation with preservation of recipient retorohepatic vena cava. This technique allows the maintenance of the caval flow during the anhepatic phase and the avoidance of the veno-venous by-pass. The liver is detached completely from retrohepatic vena cava with the legation of accessory veins. The main hepatic veins are clamped and the liver removed. A temporary porto-caval shunt to avoid splancnic congestion has been carried out only in 6% of the cases. The upper vena cava of the graft is anastomized end to end with the stump of the middle-left hepatic vein, and in 2% of the cases end to side with the recipient vena cava. The lower vena cava of the graft is stapled and the portal vein anastomosis is performed. The piggy-back technique has been performed in 50/240 of our liver transplantation and actually is our preferable technique. It is higly advisable in the case of small graft, such as reduced size liver graft, when the patient has a prior porto-caval shunt. A particular indication is fulminant hepatitis, when the technique can be employed as a bridge procedure before the implant of the graft. A further advantage is economical, since the veno-venous by-pass is not necessary.

### V022

### ARTERIAL RECONSTRUCTION DURING HEPATECTOMY FOR HEPATIC HILAR TUMOR

S. Miyakawa, A. Horiguchi, K. Miura.
Second Department of Gastroenterological Surgery, Fujita Health University School of Medicine, Toyoake, Japan

The hepatic arteries run in contact with the bile duct, and then are often infiltrated by cancers originating from the upper or hilar portion of the bile duct or from the gallbladde. Such cases have been traditionally cosidered unresectable when both right and left hepatic arteries were invaded, or required massive resection of the liver when the right hepoatic artery was invaded. If liver segmentectomy can be performed with resection and reconstruction of the segmental hepatic artery, it may be possible to preserve the potency of the liver. We succeeded in central bisegmentectomy with concurrent caudate lobectomy by using the right gastroepiploic artery for reconstruction of the posterior segmental branch of the right hepatic artery. This was performed on a patient with gallbladder cancer which had infiltrated the right hepatic artery up to the bifurcation site of its anterior and posterior branches. The right gastroepiploic artery was dissected free together with its surrounding fatty tissues along the greater curvature of the stomach. The dissection was carried down to the pyloric ring on the right and up to the last bifurcation site on the left. The right gastroepiploic artery was anastomosed to the posterior branch of the right hepatic artery in end-to-end fashion using 7-0 monofilament-nylon. Celiac angiography was conducted three weeks postoperatively. This study confirmed a patent hepatic segmental arterial anastomosis.

### V023

### RIGHT TRISEGMENT PORTAL VEIN EMBOLIZATION

M. Nagino, Y. Nimura, J. Kamiya, S. Kondo, M. Kanai, Y. Goto
1st Department of Surgery, University of Nagoya, Nagoya, Japan

We have developed embolization of the right portal vein plus the left medial portal branch (right trisegment portal vein embolization) through the "ipsilateral" approach. Its practical technique is presented in this video. The patient was a 61-year-old male with hilar cholangiocarcinoma. Two percutaneous transhepatic biliary drainage catheters were placed to drain the entire biliary system and to evaluate the cancer extent. Based on the preoperative findings of several diagnostic imagings, right hepatic trisegmentectomy with *en bloc* resection of the caudate lobe and the extrahepatic bile duct was scheduled. However, the volume of the left lateral segment, assessed by volumetric study using computed tomography, was only 249 $cm^3$ (the percentage of the liver resected was 81%). Thus, portal vein embolization was performed to increase the safety of liver resection. Two kinds of 5.5F triple-lumen balloon catheters (types I and II), designed at our facilities, were used for embolization. The type I catheter has one lumen connected to the balloon and two other lumens connected to the catheter tip. The type II catheter is similar to the type I catheter except that the two lumens open just proximal to the balloon. Under fluoroscopic control the type I catheter was advanced into the portal vein through a 7F catheter sheath introduced through an ultrasonogram-guided puncture of the right anterior portal branch. The left medial portal branch was first embolized by using this catheter. Because this catheter is made of polyethylene, it could be curved by steam and advanced easily into the left medial branch. Then the right posterior inferior (P6) and the right posterior superior (P7) portal branches were embolized individually by the same catheter, because in this case P6 and P7 branched off at the portal bifurcation independently. After exchanging the catheter, the right anterior portal branch was embolized by the type II catheter. Fibrin glue mixed with iodized oil was used as the embolic material. The volume of the left lateral segment increased to 291 $cm^3$ two weeks after the embolization. Right hepatic trisegmentectomy with caudate lobectomy was carried out as scheduled. Posthepatectomy hyperbilirubinemia occured but subsided. The patient has now been well without any signs of recurrence 15 months after the surgery. In conclusions, because right hepatic trisegmentectomy for hilar cholangiocarcinoma results in extensive resection of functional liver, right trisegment portal vein embolization is advisable as preoperative management to increase the safety of extensive liver resection Trisegment embolization is achievable only through the ipsilateral approach. This approach is safer because the portal branch designed for embolization is punctured, and consequently the catheter sheath can be removed soon after embolization.

*[The length of the video: around 10 minutes]*

### V024

### EXTENSIVE HEPATOBILIARY RESECTION WITH PORTAL VEIN RECONSTRUCTION FOR PRIMARY MALIGNANT LYMPHOMA OF THE LIVER

Y. Nimura, J. Kamiya, S. Kondo, M. Nagino, M. Kanai
The First Department of Surgery, Nagoya University School of Medicine, Nagoya, Japan

We will demonstrate preoperative imagings and an operative procedure of a left hepatic trisegmentectomy with extrahepatic bile duct resection and portal vein reconstruction with intraportal tumor thrombectomy for a rare case of advanced primary malignant lymphoma of the liver originated from the segment 1 and 2 which diffusely involved the biliary structure at the hepatic hilus from the left to the confluence of the right anterior and posterior segmental branches. CT and portography showed marked tumor thrombus which obstructed the left intrahepatic portal branches and the right anterior segmental branch and separated the portal bifurcation of S6 and S7 branches. Percutaneous transhepatic biliary drainage (PTBD) was performed in the right anterior and posterior segmental ducts and the operation was carried out on July 29, 1993.

At laparotomy, a clear demarcation was observed at the right portal fissure and collateral portal circulation was found around the bilairy ducts. After dividing the left and middle hepatic arteries, the portal vein was extensively exposed up to the bifurcation and tumor thrombectomy was performed through the longitudinal venotomy on the main portal vein extended up above the bifurcation while preserving the portal blood flow through the above collateral veins. After removing the portal tumor thrombi, the venotomy was closed longitudinally and the left and right anterior portal vein were ligated. After obtaining the portal blood flow into the right posterior segment, the common bile duct and the collolateral veins were resected above the pancreas, and the liver was transected along the demarcation on the right portal fissure and the caudate lobe was also resected en bloc. The right posterior segmental duct was resected at the confluence of S6 and S7 ducts and reconstructed with a jejunal loop.

Postoperative histological examination revealed primary malignant lymphoma of the liver: diffuse large cell, B type. The patient, 55 year-old female, received postoperative chemotherapy and has been living quite well without tumor recurrence for 2 years and 5 months.

### V025

**EXTENDED LEFT HEPATECTOMY WITH CAUDATE LOBECTOMY FOR A LARGE CAUDATE TUMOR**

G. Nuzzo, F. Giuliante.
Geriatric Surgery Unit, Catholic University, Rome, Italy.

Surgical resection of caudate tumors often requires an associated right or left hepatectomy depending upon the extention of the mass. The video shows the case of a 31 year old female who presented with abdominal pain and a history of oral contraceptives assumption, and was found to have a 10 cm. tumor in the caudate lobe revealed with abdominal US. Subsequent hepatic CT scan and MR documented that the tumor was closely related to the median and left suprahepatic veins at their confluence with the vena cava. A fine needle biopsy diagnosed the adenomatous nature of the lesion. At surgery, after the mobilization of the left liver, the tumor extended to the left side of the hilum. Operative sonography was used to exactly delineate the lesion and its vascular relations. The dissection of all draining veins of the caudate lobe was pursued under direct vision over the anterior surface of the vena cava, but the control of the major hepatic veins for an isolated resection of segment I resulted highly dangerous and an anterior approach to the caudate lobe through a left hepatectomy was preferred. The right lobe was mobilized and the retrohepatic vena cava was skeletonized from the renal confluence to the diaphragm with section of the right adrenal vein. The portal dissection was done and the left artery, left portal branch and left biliary duct were transected. After pedicle clamping (PC), parenchimal transection was carried out using a kelly clamp. Before reaching the caval plane and after hemodynamic tolerance test, hepatic vascular exclusion (HVE) was accomplished associating to the PC, the clamping of the vena cava above and below the liver. PC lasted 55 minutes and HVE time was 40 min. No transfusion was required. Postoperative recovery was uneventful with discharge home on the 10th hospital day. Histology revealed focal nodular iperplasia.

### V026

SMALL-CALIBRATED MESOCAVAL INTERPOSITION SHUNT (SCMCIS) IN CIRRHOTICS WITH SCLEROTHERAPY FAILURE - STANDARD METHOD OF CHOICE ?

K.-J. Paquet, A.Lazar, R.Kuhn, Depts. of Surgery und Medicine, HEINZ KALK-Hospital, D-97688 Bad Kissingen, Germany

Introduction: 1972 DRAPANAS introduced a high lumen mesocaval interposition shunt with the 18-20mm DACRON prothesis to treat bleeding esophageal varices. This new type of operation was well accepted because its technical simplicity, efficacy to prevent esophageal hemorrhage and low rate of encephalopathy. In the later course a rate of anastomotic thrombosis up to 30% and encephalopathy up to 20% was described restricting its indication. Patients and Methods: Since Jan. 1, 1987 our group is using a 10mm PTFE (Goretex) -prothesis prospectively. The results of consecutive 96 pat. failing endoscopic sclerotherapy (ES) up to the 1st of Jan. 1995 are described. In 97% liver cirrhosis, mostly alcoholic origin was the underlying disease. 51 pat. belonged to the CHILD-PUGH classification A, 41 to B and 4 to C (emergency shunts). 58 men and 38 women had a median age of 56.3 (21-72) years. Results: There were 8 hospital deaths, 2 of the emergency group, mainly due to liver failure; thus, the total hospital mortality is 8.3% and 6.6% of the elective shunt. 4 postoperative thromboses (4.2%) developed and 5 recurrences of hemorrhage managed by ES. Preservation of postoperative portal perfusion was demonstrated in all cases. Encephalopathy rate was 9% easy to manage by diet and lactulose. There were 12 late deaths up to Jan. 1, 1995 (13.6%). Thus, the five and eight years survival time according to KAPLAN-MEIER is 75%. Conclusion: The results show, that SCMCIS has a place in the treatment of portal hypertension, particularly in ES-failures. It is an excellent alternative choice to the distal splenorenal shunt and does not interfere with later liver transplantation.

### V027

RIGHT HEMIHEPATECTOMY AND SMALL BOWEL RESECTION FOR CARCINOID SYNDROME (USING WATER JET DISSECTION).

Partensky C. Service de Chirurgie Digestive. Hôpital E. Herriot. 69437. Lyon. France.

A 68 year-old woman complained of carcinoid syndrome with severe diarrhea and flush. The primary tumor was located at the ileum, while large necrotic metastases were present in the right lobe of the liver.

A right hemihepatectomy with concomitant small bowel resection was performed.

A Swan-Ganz catheter was inserted preoperatively and continuous infusion of Somatostatin was started at the beginning of the procedure.

Liver resection was the first step of the procedure. Water jet dissection was used for dissection of the hepatic pedicle and removal of one large metastatic lymph node, and for selective dissection of the hepatic parenchyma under portal clamping. It was used also at the second step of the operation i.e. small bowel resection, for selective dissection of the mesenterium and removal of lymph nodes satellite to the primary tumor.

The patient underwent an uneventful recovery with complete regression of the carcinoid syndrome.

Carcinoid syndrome can be cured by concomitant resections of the primary tumor and of liver metastases when both lesions are resectable.

VHS PAL 8 mn 30 sec.

### V028

**Hydatid disease of the liver: cystopericistectomy**

**Rovati V., Nervetti G., Faleschini E., Colturani F..**
**Department of Surgery, University of Milan, Milan, Italy**

**The film shows a case of a voluminous echinococcus cyst localized in left liver and appearing on the surface of the parenchyma. Pericistectomy with intact cyst wall was performed; vascular exclusion, in order to avoid blood leakage, was obtained using Pringle's manouvre.**
**This treatment has the better outcome because it doesn't cause reduction of the functioning parenchyma and has better possibilities for radical:**
**1) parasite exclusion,**
**2 ) removal of external vesiculation,**
**3) relapse prevention,**
**4) complications prevention**

### V029

REGULATED RIGHT HEPATECTOMY FOR CHOLANGIOCARCINOMA.

R.RUGGIERO,R.REA,A.FERRARA,G.VIOLA
INSTITUTE OF EXPERIMENTAL SURGERY,SECOND UNIVERSITY OF NAPLES,NAPLES,ITALY

Cholangiocarcinomas,more uncommon than hepatocellular tumors allow in the majority of cases a very poor prognosis thous influenced by various factors linked to the neoplasia.In the most differentiated histological forms,with structures that looks like the ducts of Hering,to which rather low levels of AFP are associated,the expectation of life is clearly better,compared with undifferentiated forms(35vs4 months of average survival).A right hepatectomy is presented(V,VI, VII,VIII segment)carried out for a well differentiated cholangiocarcinoma,in a non-carrier of Australia antigen with an AFP rate of 115ng/ml. The decision for resection was taken on the basis of a preoperative angiographic and morphological study(ECO,TC,lapatoguided biopsy),also through the extemporary valuation of the stage and grading of the neoplasia.The presence of a single mass on the periphery of the right lobe,the lack of satellitosis,the good quality of the healthy hepatic parenchyma, the resection with adequate margin free of neoplasia,confirmed by the intraoperative ecography,led us to consider the regulated resection for curative purposes,technically possible and more advantageous for the life expectation of the patient,compared to a conservative treatment.After 6 months the patient was in good healt.

### V030

MINIMALLY INVASIVE HEPATIC RESECTION USING LAPAROSCOPY AND MINITHORACOTOMY

M. Sato, Y. Watanabe, S. Ueda, S. Iseki, N. Sato, S. Kimura
Department of Surgery II, Ehime University School of Medicine, Ehime, Japan

There is few report about the feasibility of minimally invasive hepatic resection even in the laparoscopic era. We introduced a new technique of hepatic resection using laparoscopy and minithoracotomy for peripheral tumor in the right lobe. The patient was a 61-year-old man having HCC associated with liver cirrhosis due to hepatitis C. The tumor, 40 mm in diameter, was located in segment VI. He had impaired hepatic function and decreased platelet count (indocyanine green clearance rate, 0.106; and platelet count, 65000 /mm$^3$).

Procedure: The ligamental attachments of the right hepatic lobe was divided under the guidance of laparoscopy. Laparoscopic sonography revealed a small satellite, 11 mm in diameter, in segment IV. Following a skin incision of 12 cm in length, anterior thoracotomy not extending to the abdominal wall was made. The right thoracic cavity was entered through the 8th intercostal space. After dividing the diaphragm and the costal arch, the right hepatic lobe was exposed. The satellite nodule was coagulated with microwave. Parenchymal dissection was carried out using an ultrasonic surgical dissector and electrocautery without holding the liver, occluding the inflow, or dissecting the hilum. There was a slight bleeding tendency; the blood loss was 1700 ml. The resected liver volume was 135 g. The patient recovered uneventfully.

Results: This procedure provides minimal access to the dissecting plane of the liver and enables non-touch resection without vascular occlusion. The procedure of parenchymal dissection was a little demanding in terms of bleeding control due to difficulty to compress the liver. Patients having peripheral tumors in the right lobe are candidate for this technique. This might prevent intraperitoneal adhesion which disturbs the repeated surgical treatment for recurrent HCC.

Conclusion: Introduction of laparoscope assisted surgery and minithoracotomy opens the door to a new era of minimally invasive surgery in hepatic resection.

(12 min.)

### V031

NARROW-LUMEN MESOCAVAL PTFE FLEX INTERPOSITION SHUNT WITH FIBRIN SEALANT IN CIRRHOTIC PATIENT WITH ASCITES - SAFE PROCEDURE

Severtsev A., Ivanova E. Medical Centre for Russian Government (MCRG); Dpt. of Surgery, Hospital #51, Moscow, Russia

Ascites in patients with liver cirrhosis signals a decompensation in the ability of the organism to live in simbiosis with its disease. The serious problem is diuretic-resistant ascites (DRA). The number of different treatment options for ascites treatment is inversely proportional to the overall success rate in terms of palliation or cure.

In our Clinic we used 2-stages surgery for the patients with DRA. At the 1st stage we use peritoneo-venous shunt (PVS) to normalize nutritional status of patients and then a H-mesocaval interposition shunt of narrow-lumen (2nd stage). We treated 3 patients and all patients were in good condition during next 12 month after the beginning of treatment.

The video presents an operation of narrow-lumen (up to 10 mm) H-mesocaval interposition shunt with PTFE vascular graft (Impra, USA) with the support of vascular anastomoses by fibrin sealant (Tissucol, Immuno, Austria) for patient with portal hypertension complicated with the DRA (spironolacton-resistant). This operation was performed after previous (3 months before) peritoneo-venous shunting (Denver, USA) and followed by normalization of nutritional status. The use of preliminary PVS, PTFE Flex Prothesis and fibrin sealant gave exellent possibilities to have safe main surgery and good long-term results.

### V032

**UNCOMMON BEHAVIOR OF THE HYDATIC DISEASE OF THE LIVER**

P.J.Sisco, N.B.Perrone, G.F.Pagliarino
Surgical Division, Pirovano Hospital, Buenos Aires, Argentina

This is the case of a patient of 52 years of age, male, with upper right abdominal pain, weight loss, asthenia, and light jaundice. The ultrasonography and C.T. scan showed the typical image of a 10 cm diameter hydatic cyst of the liver, in the right lobe, with involvement of the diaphragm, and secondary vesicles inside. During the hospitalization jaundice increased abruptly, without cholangitis, which made an early surgical treatment necessary. A bilateral subcostal incision prolonged to the sternum was performed. During the hilus hepatic dissection, involvement of the duct junction and portal bifurcation was evident, therefore a concomitant malignant pathology was suspected. Intraoperative biopsy of adenomegaly was made without evidence of metastasis. The pathology was treated radically, and the surgical procedure was right hepatectomy and the resection of : extrahepatic bile duct, bile duct juntion, portal bifurcation, partial right diaphragm, with anastomosis of the portal vein to the left portal branch, and the left hepatic duct to the jejunum. Operative time was 10 hours. Immediate evolution was satisfactory but the patient needed hemodialysis. Histopathological report of the specimen was: " Hydatic cyst with surrounding fibros tissue extending from the peripheral cyst to the hepatic hilus with involvement of the bile duct junction and portal bifurcation ". Conclusions : This case confirms what we think about this pathology. We consider it a benign disease with malignant behavior since it involves structures, replaces the parenchyma, makes metastasis and impaires the patient's general state.

Video length : 15 minutes.

### V033

RIGHT HEPATIC RESECTION IS NOW ONE-HOUR OPERATION WITH NO BLOOD TRANSFUSION

Ken Takasaki, M. Tsugita, T. Ohtsubo, M. Yamamoto, A. Fujimoto*, S. Katagiri, K. Akiyama
Institute of Gastroenterology Tokyo Women's Medical College
*Kenou Gastroenterological Hospital

We have experienced 1,000 cases of hepatic resection for primly liver cancer for 20 years, while we are making effort to simplify the operative procedure of hepatic resection.

We devised Glisson's sheath pedicle transection method as hilum vascular preparation. In this procedure, portal triads (hepatic artery, portal vein, bile duct) are transected not one by one but as one bundle. Transection of right hepatic vessels which flow into the liver from hepatic hilum is completed within only several minutes. For the procedure of hepatic parenchymal dissection, we developed anterior approach, which does not require mobilization of right hepatic lobe from diaphragmatic attachments. Since this procedure does not require the blind dissection, at the back of the liver, the risk of bleeding and some other trouble are reduced especially in cases of big tumor.

Right hepatic resection in our procedure can be done within 500ml of blood loss during operation, and it takes about one hour.

### V034

**RETROHEPATIC AND INFRARRENAL INFERIOR VENA CAVA RESECTION WITH GRAFT REPLACEMENT.**

**Emilio Vicente M.D., Jose Mª Egaña M.D., Javier Nuño M.D., Manuel Devesa M.D. Alberto Honrubia M.D. Angel Candela M.D. Gemma Monge M.D. Luis Fogué M.D.**

Vena cava resecction with graft replacement is a procedure rarely performed in the surgical practice. The majority of these resections have been partial, without the need for a prosthetic replacement. At the present time, technical modifications, technological innovations and the contribution of different experienced specialists, allow the performance of this procedure with low morbidity and mortality rates.

This video details the presentation and course of a patient with infrarenal and retrohepatic IVC neoplastic thrombosis secondary to renal cancer, not available to thrombectomy because of infiltration of the caval wall. He underwent a inferior cavectomy from hepatic veins to iliac bifurcation and replacement with a large expanded polytetrafluoroethylene graft (ePTFE) 20 mm. in diameter and 28 cm. in length. A groin arteriovenous fistula was created between superficial femoral artery and saphenous vein.

Sixteen months after the operation, the patient remains alive, without evidence of recurrence or graft occlusion.

To our knowledge, our patient is the first case of sucessful and uncomplicated replacement of the retrohepatic and infrarenal IVC for caval thrombosis secondary to renal cancer with a large synthetic graft and not undergoing liver resection.

### V035

**LIVER TRANSPLANTATION FOR GIANT CAVERNOUS HEMANGIOMA OF THE LIVER: AN UNUSUAL SURGICAL OPTION**

**Emilio Vicente M.D. Javier Nuño M.D. Victor S. Turrión. Jesús Igea M.D. Yolanda Quijano M.D. Fernando Pereira M.D. Nicolás P. Mora M.D. Julian Ardaiz M.D.**

**Liver Transplantation Unit. Hospital Ramón y Cajal. Clínica Puerta de Hierro. Madrid. Spain**

Cavernous hemangioma is the most common benign tumor of the liver. Most of them are small and asymptomatic and are occasionally found at ultrasound study or laparotomy.

Giant cavernous hemangioma of the liver, although rare are more prone to spontaneous rupture or hemorrhage. Hepatic fibrosis is a rare alteration in these patients, but may occur when hepatic venous outflow obstructionis present.

The video shows a liver transplantation procedure for giant cavernous hemangioma of the liver (maximum diameter: 30 cm) associated to chronic Budd-Chiari Syndrom. The lesion was located in the right lobe and the medial segment of the left lobe.

The video include perioperative imaging techniques and details of the surgical technique.

### V036

HEPATIC 8 SEGMENTECTOMY USING ENHANCED INTRAOPERATIVE ANGIOECHOGRAPHY WITH GLISSON SHEATH CODE TRANSECTION AT THE HEPATIC HILUS

H. Yasuda, T. Takada, T. Uchida, T. Isaka, Y. Toyota
First Department of Surgery, Teikyo University
School of Medicine, Tokyo, Japan

Segment 8 hepatic resection is presented by video, using enhanced intraoperative angioechography with Glisson sheath code (portal vein, bile duct, and hepatic artery) transection at the hepatic hilus. In this procedure, the presence or absent of tumors and satellite nodules was detected by the use of enhanced intraoperative angioechography using $CO_2$ gas at the ramus in the anterior segment after taping Glisson sheath code in the right lobe. After confirming tumor-bearing Glisson sheath code, ICG (Indocyanine green) was injected into portal vein which feeds the tumor, and region stained green with ICG. Perform resection of upper anterior segment in the liver so that the region stained with ICG can be resected from the liver surface towards the hepatic hilus after severing the Glisson sheath code which feeds a tumor using hilar approach.

This procedure for carcinoma is important not only to reduce blood loss, but also to prevent the intraoperative dissemination.

### VIDEO PRESENTATIONS

### Topic: PANCREAS

### V037

RESECTION OF THE INFERIOR HEAD OF THE PANCREAS

T.Asano, T.Nakagohri, W.Takayama, S.Kobayashi,T.Uematsu, T.Kenmochi, S.Okazumi, O.Kainuma, T.Kubota, C.Iwashita, Y.Sugamoto and K.Isono
Department of Surgery, Chiba University, School of Medicine

We developed a new partial resection of the head of the pancreas with an end-to-side pancreaticoduodenostomy (or pancreaticojejunostomy), while preserving the duodenum, the common bile duct and upper part of the head of the pancreas around the duct of Santorini.

**Patients**

Inferior pancreatic head resection was performed for three patients with intraductal papillary adenoma or non-invasive adenocarcinoma with mucin hypersecretion in the head of the pancreas. They are all male, and their ages were 60, 74, 75 respectively.

**Operative procedure**

The head of the pancreas is approached through a midline upper abdominal incision. A Kocher maneuver is not performed, for protecting the mesoduodenum and vessels to the duodenum. Size and location of the primary lesion is evaluated with intraoperative ultrasonography. Invasion of adjacent structure is also investigated. An important step of the operative procedure before the resection is the tunneling of the pancreas and the dissection of both the superior mesenteric vessels. Care must be taken to preserve arterial blood supply to the duodenum. Only pancreatic branches of the inferior pancreaticoduodenal vessels should be carefully ligated and divided to avoid injuring the inferior pancreaticoduodenal vessels along the entire length of the duodenum. The management of these small vessels is one of the most delicate steps in this resection. Division of the pancreas begins at the inferior margin of the neck of the pancreas toward the common bile duct. During division, bleeding points are transfixed with sutures of 5-0 nylon. It is important to place four or more sutures of 4-0 absorbable monofilament in the distal duct of Wirsung for preparing pancreaticoduodenostomy. The common bile duct is well exposed after the excision. The duct of Wirsung is anastomosed to the 3rd portion of the duodenum (or jejunum) in an end to side fashion. Mucosa-to-mucosa stented pancreaticoduodenostomy (or pancreaticojejunostomy) is performed with 4-0 or 5-0 absorbable monofilament sutures.

**conclusion**

We believe resection of the inferior head of the pancreas with an end-to-side pancreaticoduodenostomy has a significant role to play in the management of patients with benign diseases and localized malignant tumors of the pancreas.

The length of the video is 10 minutes.

### V038

A NEW METHOD OF TREATING EXTERNAL PANCREATIC FISTULAE.

I.Buriev,A.Karelin,T.Savvina,V.Prizov,L.Medov V.Kobrin,M.Ponamareva.

A.V.Vishnevsky Institute of Surgery, Moscow Russia.

An experience of the treatment of 95 patients with primary external (63) and purelent pancreatic (32) fistulae by occlusion of thy fistulous tract,segment of the mainpancreatic duct (MPD) and parapancreatic collections is presented. The fistulae developed following abdominal trauma (21%),pancreonecrosis(66,4%) or operations on intraabdominal organs(12,6%) The fistulae were grouped as primary terminal (46),lateral (17) and purulent-pancreatic(32) depending on their relationship with the MPD. The diagnosis of fistulae was based on findings US, fistulography, level of amylase and microbial bodies in the fistulous discharge. Occlusion was performed under roentgenologic control using "RABROM"-an absorbable antibacterial biologic occlusive contrast medium, created on fibronectin base.The medium has a resultant density of 0,05 Pa.,and polymerisation time of 2-4 mins. Postoperatively patients received "Sandostatin" in dose of 0,1 mg four days. A cure rate of 77,9% (84 patients) was achieved,recurrence of fistulae were observed in 11 patients and now deaths occured. Patients were on admission for 5-7 days,operation lasted for not more than 10 mins.

Occlusion of pancreatic fistulae is an effective,less invasive, economically beneficial method of treating patients.

length of vidiofilm 10 mins

### V039

**OPEN TREATMENT OF SECONDARY PANCREATIC INFECTIONS**

**F. Crucitti, GB. Doglietto, S. Alfieri, F. Pacelli, G. De Vivo**
**Department of Surgery, Catholic University, Rome, Italy.**

**BACKGROUND The surgical management of secondary pancreatic infections is still controversial.**

**AIM The video shows the surgical approach and the technique adopted in our unit.**

**METHODS Surgical technique consists of abdominal entrance by a bilateral subcostal incision and exposure of the pancreas by dividing the gastrocolic omentum. Accurate pancreatic necrosectomy or abscess debridment is performed preserving the vital parenchyma; at the end of the operation the abdomen is left opened and a marsupialization of the lesser peritoneal sac is created by suturing the greater curvature of the stomach and the transverse colon respective to the posterior fascia of the superior and inferior edges of the laparostomy. In this way, a cavity is created trough which the pancreas is exposed. The cavity is entirely packed with gauze. Multiple silicon-rubber drains are placed in the subphrenic spaces, pouch of Douglas and in the pancreatic bed. The abdominal incision is left either completely open or partially closed laterally with bridges allowing the entire hand to enter the laparostomy.**

**CONCLUSIONS In the surgical treatment of secondary pancreatic infections our policy is to perform the open-packing technique that use laparostomy and marsupialization of the lesser sac. This technique allows for complete drain externally from the pancreatic compartment and, at the same time, separates the pancreatic compartment from the rest of the peritoneal cavity.**

### V040

**SUBTOTAL DUODENOPANCREATECTOMY AND INTRAOPERATIVE RADIOTHERAPY FOR TREATMENT OF RESECTABLE PANCREATIC HEAD CARCINOMA.**

**F. Crucitti, G. Doglietto, D. Frontera, G. Viola.**
**DEPARTMENT OF SURGERY - CATHOLIC UNIVERSITY SCHOOL OF MEDICINE, ROME, ITALY.**

Our video shows subtotal duodenopancreatectomy. This is just a part of a complex protocol for the treatment of resectable pancreatic head carcinoma, which includes:

- **subtotal** (or **total**, when needed) pylorus-sparing **pancreatic resection** (according to Traverso-Longmire technique);
- **regional lymphectomy** extended to hepatic hilus, common hepatic artery, celiac trunk, splenic vessels, mesentericoportal trunk and interaortocaval space;
- **intraoperative radiotherapy (IORT** - 10 Gy), delivered trough an electron beam (6 MeV) to an area including the portal vein, the splenomesenteric confluence, the superior mesenteric vein up to its branches, the celiac trunk, the common hepatic artery, the proximal splenic vessels, the suprarenal aorta and caval vein;
- the subsequent execution of end-to-side **pancreatico-jejunostomy, hepatico-jejunostomy** and **duodeno-jejunostomy**, on a single intestinal loop;
- an **external beam radiation therapy (EBRT** - 50 Gy), delivered using a photon beam (9 MV), about 4 weeks after surgical resection, to a target volume including tumoral and lymphatic bed.

Twenty-two patients were treated following the above mentioned protocol: we obtained a good local control (90 %), but this result was not matched with a satisfactory long-term survival (median: 11.8 months) due to the high incidence of metastasis (67 %).

Thus, new therapeutic modalities should be tested; in this respect, neoadjuvant radio-chemotherapy seems to be the most promising.

### V041

**PANCREATICODUODENECTOMY WITHOUT PANCREATIC-JEJUNOSTOMY**

G. De Sena - F. Chianese - F. La Rocca - G. Picardo - P. Festa
Dept. of Surgery - "San G. Moscati" Hospital - Avellino

It is an operative technique performed during seven pancreaticoduodenectomy without pancreatic-jejunostomy.

The pancreatic-duct is closed with any human fibrin glue introducted with a special catheter at two way and a very-low absorbable suture placed at "U".

The biological glue is used also for the impermeabilization of residual section of pancreas.

There was no postoperative complications and no perioperative deaths. All patients left the hospital within 2 weeks of surgery.

The median follow-up time was 24 months and no one patient showed symptoms of pancreatic failure.

### V042

LONGITUDINAL WIRSUNG-JEJUNAL ANASTOMOSIS BY MERCADIER

S.Dešković, M.Martinac, M.Bezjak, L.Patrlj, B.Kocman, B. Makaruha, S.Jadrijević, Ž.Posarić, T.Šoša.
MERKUR UNIVERSITY HOSPITAL, ZAGREB
Department of Surgery,
Zagreb University School of Medicine

Longitudinal anastomosis of Wirsung's channel and jejunum was first used by Cattel as a palliative operation in cancer of the pancreatic head. Mercadier inaugurated a similar procedure in surgical treatment of chronic pancreatitis. The fundamental condition requiring such an operation is dilated pancreatic channel of 8-10 mm in diameter. Good longtime results of this operation consist in absence of pain, good drainage of pancreatic secretion into the jejunum and the achivement of the complete function of the gland.

The video-film presents our technique of operation in its all details. It consists of identification of Wirsung's channel, its intraoperative, radiograph presentation, longitudinal transparenchymn discission of the channel in length of 8-10 cm, and performance of latero-lateral Wirsung jejunal anastomosis with an isolated winding of the jejunum by Roux. Anastomosis is stitched in two layers with slow-resorbed atraumatic material.

We are si far satisfied with our results, because longtime good results have shown in almost 80% of operated patients. As the clinician knows well, patients with chronic pancreatitis have persistent difficulties followed by recidivous attacs of pancreatitis with all its complications. When the indication is well set up and conditions completed, we consider latero-lateral longitudinal Wirsung-jejunal anastomosis a method of choice in the surgical treatment of patients with chronic pancreatitis. Its acceptable postoperative morbidity and mortality, undisturbed endocrine function of the gland, according to our results, gain an advantage over the resection methods of treatment of chronic pancreatitis.

### V043

**LAPAROSCOPIC SIDE-TO-SIDE PANCREATICO-JEJUNOSTOMY (PUESTOW) FOR CHRONIC PANCREATITIS.**

M. Gagner, MD. Dept of General Surgery, The Cleveland Clinic Foundation, Cleveland, Ohio.

A 37 y.o. male with hereditary primary hyperparathyroidism has developed calcified chronic pancreatitis with a dilated pancreatic duct.

He underwent a laparoscopic cholecystectomy and drainage of the pancreatic duct. A total of six trocars were used in the umbilicus and subcostal area (three 10 mm trocars and three 5 mm trocars). Following cholecystectomy and cholangiogram, the gastrocolic ligament was opened under the gastroepiploic arcade. A laparoscopic babcock was used in the epigastric port to retract the stomach superiorly, exposing the anterior body of the pancreas. The pancreatic duct was localized using a #22 needle percutaneously for a pancreaticogram under fluoroscopy. The duct was then opened with laparoscopic scissors and cautery. Pancreatolithiasis were retrieved with a fogarty and several forceps. Pancreatoscopy was completed using a flexible choledochoscope percutaneously. The anastomosis was then made with running 2-0 aborbable sutures with the antimesenteric side of the proximal jejunum (side-to-side) antecolic using a two handed technique. Two drains were left close to the anastomosis in the lesser sac.

Laparoscopic pancreaticojejunostomy is a potential alternative to open surgery in selected patients (10 min.).

### V044

**LAPAROSCOPIC TRANSDUODENAL SPHINCTEROPLASTY FOR PAPILLARY STENOSIS AND RECURRENT CHOLANGITIS.**

M. Gagner, MD, G. Breton, MD, Dept of General Surgery, The Cleveland Clinic Foundation, Cleveland, Ohio, and Hotel-Dieu de Montreal, Montreal, Quebec.

The patient had recurrent episodes of cholangitis and required several sphincterotomies by ERCP for papillary and distal bile duct stenosis. After subsequent failures, a sphincteroplasty was indicated. The video describes the operative laparoscopic technique. Four trocars are inserted in the right flank and right paramedian areas in a semicircular line away from the second duodenum. An angulated 10 mm 30° laparosope was used for optimal view. A proper Kocher maneuver is performed to mobilize fully the duodenum. Suspensory sutures passed through the abdominal wall are positioned on the upper and lower part of the duodenum. A longitudinal duodenotomy of 5 cm long is made, a stay suture is positioned on the ampulla for traction and better exposure.

The sphincterotomy is performed after cholangiogram at 11 o'clock using laparoscopic straight scissors with cautery. This sphincteroplasty is achieved by interrupted suture with 4.0 monofilament absorbable sutures with intracorporeal knots between the bile duct and the duodenal wall with a two handed technique. Closure of the duodenotomy is performed with a running suture followed by periduodenal drainage. The patient was discharged on the fourth postoperative day without any leakage or complications. Laparoscopic transduodenal sphincteroplasty for papillary stenosis is an alternative to open surgery.

### V045

**LAPAROSCOPIC RESECTION OF AN INSULINOMA.**

M. Gagner, MD, M. F. Herrera, MD, E. Prado, MD, Department of General Surgery, The Cleveland Clinic Foundation, Cleveland, Ohio and Instituto Nacional de la Nutricion Salvador Zubiran, Mexico City, Mexico.

Islet cell tumors of the pancreas are rare, but benign lesions can be treated with an open enucleation technique. This video describes completely the technique of laparoscopic pancreatic enucleation of an insulinoma on the anterior surface of the pancreas at the junction of the body and tail.

A 25 y.o. male with neuroglycopenic symptoms for 9 months was more symptomatic after periods of fasting and was ameliorated with sweet beverages. The fasting glucose was 54 mg/dl (N=70-110), fasting insulin was 79.4 micro units international (N=0-30) and fasting "C" peptide 12.8 mg/dl (N=0.8-4). The angiogram revealed a blushing tumor compatible with insulinoma (near the tail).

Four trocars are inserted in the left upper quadrant with the patient in a semi-lateral position (45°). The gastrocolic ligament is entered for exposure of the anterior body of the pancreas. A cephalad retraction of the greater curvature of the stomach is essential. The insulinoma is carefully dissected from the normal pancreatic parenchyma using a hook dissector with cautery. The vessels behind the insulinoma are ligated with titanium clips, extraction is performed via one of the ports using an opaque laparoscopic bag. A drain is left over the pancreas behind the stomach. The patient left the hospital on the fourth post-operative day without any complications and is free of recurrence after a follow-up of nine months. Laparoscopic enucleation of insulinomas is a very good alternative to open surgery.

### V046

**LAPAROSCOPIC PROXIMAL AND DISTAL PANCREATIC RESECTION.**

M. Gagner, MD, A. Pomp, MD. Dept of General Surgery, The Cleveland Clinic Foundation, Cleveland, Ohio and Hotel Dieu de Montreal, Montreal, Quebec.

Patients who had proximal and distal laparoscopic pancreatic resection performed were followed and evaluated for morbidity and recurrence.

Since January 1992, 16 patients underwent an attempted laparoscopic Whipple (8) or distal pancreatectomy (8). The indications for laparoscopic Whipple were pancreatic adenocarcinoma (3), chronic pancreatitis (2), ampullary adenocarcinoma (2), and distal bile duct carcinoma (1). The mean age was 63 (range 33-82). The conversion rate was 50% with a mean operating time of 9.1 hours for laparoscopic Whipple versus 4.6 hours for converted Whipple. The hospital stay was slightly longer with laparoscopic Whipple (31 days) versus 20 days for converted Whipple. Conversions were done for technical reasons (2) and bowel distension (2). No recurrences were seen at 18 months in laparoscopic Whipple for tumors, but recurrent pain was seen in chronic pancreatitis patients. The indications for laparoscopic distal pancreatectomy included 5 insulinomas, 1 serous cystadenocarcinoma, and 2 gastrinomas. The average age was 48 (range 27-75). The conversion rate was 62% with a mean operating time of 4.5 hours for laparoscopic distal pancreatectomy versus 7.2 hours for the converted procedure. Conversions were due to metastatic disease in gastrinomas (2), and inability to localize an insulinoma (1). The hospital stay was shorter for laparoscopic distal pancreatectomy (4.5 days) versus 12 days for open distal pancreatectomy. After a follow-up of 18 months of the four insulinomas and one cyst adenocarcinoma, there was no recurrence.

Although the series is small, no benefit was seen from a complete laparoscopic Whipple procedure. Laparoscopic distal pancreatectomy is technically easier and seems to benefit patients by decreasing their hospital stay without recurrent disease.

### V047

EXTENDED RESECTION COMBINED WITH INTRAOPERATIVE RADIATION FOR PANCREATIC CANCER

T. Hiraoka, K. Kanemitsu, Y. Kamimoto, S. Kawamoto, T. Morisaki, K. Tabaru
First Department of Surgery, Kumamoto University School of Medicine, Kumamoto, Japan

Since 1984, extended radical resection was combined with extended intraoperative radiation (IOR) to prevent local recurrence and provided a dramatic improvement in long-term survival compared with other approaches. We will show you this combined therapy by video.

An extended radical operation involved pancreatectomy with almost complete resection en bloc of the lymph nodes around the porta hepatis, the coeliac axis, the origin of the superior mesenteric artery and the aorta extending from the diaphragm to the inferior mesenteric artery. The inferior vena cava and aorta were also skeletonized with all tissue being removed en bloc. Following resection, including vascular reconstruction, a dose of 30 Gy of 9 - 12 MeV electrons was administered to the operatived field, including the para-aortic area from the diaphragm above to the inferior mesenteric artery below.

From 1984 to Nov. 1995, 30 patients underwent this combined therapy. Five year survival rate of all the patients including operatively dead cases was 19.2 %. That of cases with complete and incomplete clearance was 30.0% and 11%, respectively. According to the rules of TNM classification, five year survival rate was calculated; $n_0$ (n = 7), 20.8%, $n_1$ (n = 17), 25.5%, $M_1$ (LYM) (n = 3), 0%. Eighteen died of tumor recurrence. The cause of its death was almost always due to metastasis via vessels.

We think that extended pancreatectomy combined with IOR is best treatment for local disease control at present. Enhanced local control induced by this combined therapy, however, only has limited impact on overall survival, because of system disease progression, especially hepatic metastases. (10 min.)

### V048

EXTENDED PANCREATICODUODENECTOMY: A RADICAL APPROACH TO PANCREATIC CARCINOMA

C. Iacono, E. Facci, L. Bortolasi, PP. Aurola, G. Prati, G. Falezza, G. Mangiante, G. Serio
Department of Surgery, University of Verona, Verona, Italy

More than 50% of the patients that undergo Standard Pancreatico-duodenectomy (PD) presents loco-regional recurrence. This is caused by the tendency of the pancreatic carcinoma to infiltrate the retroperitoneal connective and to spread along the nerve plexus and the lymphatic channels towards the loco-regional nodes. The lymph nodes are divided in two groups: first and second level. First level group includes: anterior and posterior pancreaticoduodenal nodes, superior and inferior nodes of the head, common bile duct nodes, pyloric nodes and mesenteric nodes. Second group includes: common hepatic duct nodes, celiac nodes, superior and inferior nodes of the body, middle colic nodes, para-aortic nodes. Therefore PD is considered a radical procedure when it is associated to dissection of the retroperitoneal connective of the lymphatic and nerve plexi and of the loco-regional lymph nodes (1st and 2nd level). The pancreas is resected at level of the left margin of the aorta.

Extended PD is carried out en bloc with lymphadenectomy. In this way the celiac trunk, the superior mesenteric artery and the hilum of the liver are dissected free as well as the aortic segment between the celiac trunk and the inferior mesenteric artery. The pancreatic stump, the common hepatic duct and the stomach or duodenum are anastomosed on a single jejunal loop without any stents.

We have performed 24 consecutive Extended PDs from April 93 to December 95. Postoperative mortality rate is zero. Postoperative morbidity is similar to the 23 Standard PDs performed consecutively in the same period.

We believe that Extended PD represents a safe procedure and a valid approach to resectable pancreatic cancer.

The video shows our technique of Extended PD.

Length of the video: 14 minutes.

### V049

CENTRAL PANCREATECTOMY

C. Iacono, E. Facci, L. Bortolasi, PP. Aurola, G. Falezza, G. Mangiante, G. Serio
Department of Surgery, University of Verona, Verona, Italy

Benign tumors and uncertain malignant potential tumors located in the neck of the pancreas more than 2 cm in diameter or encased within the parenchima present some biological and technical problems when enucleoresection is not possible. In fact distal pancreatectomy and pancreatico-duodenectomy, that are usually performed for these kind of lesions, determine impairment of exocrine and endocrine function and digestive and immunologic disorders. Central pancreatectomy contrarly permits to save pancreatic parenchima, the anatomy of the upper GI tract, the biliary tree and the spleen.
The video shows the surgical technique:
- midline incision and exposition of the pancreatic surface;
- incision of the posterior peritoneum along the inferior and superior mergin of the pancreatic segment to be resected;
- the splenic artery is cleared and some minor collaterals are ligated;
- the posterior surface of the pancreatic segment involved by the tumor is isolated form the porto-mesenteric axis;
- the pancreatic segment is transected at least 1 cm from the tumor caudally and cephalically;
- the cephalic stump is sutured after elective tying of the Wirsung's duct or stapled;
- the distal stump is anastomosed end to end with a Roux en-Y jejunal loop.

We have performed central pancreatectomy in 11 cases (3 insulinomas, 2 non-functioning endocrine tumors, 4 serous cystadenomas, 1 mucinous cystadenoma and 1 solid cystic tumor). Mean diameter of the resected lesions was 2.7 cm (range 1.2-4). Mean operative time was 250 min. (range 210-300). Postoperative mortality was nil, and morbidity was very low. PancreoLauryl test and fecal fat dosage showed a normal exocrine function in all the patients. Oral Glucose Tolerance Test did not worsen after operation in anyones. All the patients are doing well at a mean follow up time of 71 months (range 16-156). Eventually central pancreatectomy represents a valid and safe procedure with low incidence of complications and a normal postoperative exocrine and endocrine function.
Length of the video: 12 minutes.

### V050

SURGICAL AND MULTIMODAL TREATMENT OF ENDOCRINE TUMORS OF THE PANCREAS

C. Iacono*, N.Nicoli*, GC. Mansueto§, G. Mangiante*, L. Bortolasi*, E. Facci*, G. Serio*
Departments of Surgery* and Radiology§, University of Verona, Italy

Endocrine tumors of the pancreas can be cured only with a surgical approach. The treatment is mandatory either for small tumors or for large tumors involving contiguous organs that are resected with the lesion. Aggressive surgical procedures are attempted also in the cases of liver metastases, removing the primary and the secondary lesions. If resection of the hepatic metastases is not possible because of multiple lesions, a multimodal treatment is adopted with resection of the pancreatic lesion and chemoembolization and/or alcoholization of the liver masses.
The surgical options depend on the size, the loco-regional extension and the location of the tumor; other important factors in the choice of the treatment are benignity or malignancy of the tumor and the presence of associated MEN.
The video shows:
- meticulous palpation of the pancreatic gland, this step is essential before planning any surgical procedures;
- exploration of the pancreas and the liver by means of intraoperative US scan;
- techniques of enucleation for insulinoma of the pancreatic head and for duodenal gastrinoma, central pancreatectomy for tumor of the pancreatic neck, left pancreatectomy and resection of the pancreatic tail preserving the spleen for 2 insulinomas respectevely of the body and of the tail of the pancreas;
- major resections en-bloc with contiguous organs, as pancreatico-duodenectomy with resection and reconstruction of the mesenteric-portal axis for a non-functioning endocrine tumor.

The video moreover faces the management of endocrine tumors of the pancreas with sinchronous or metachronous liver metastases. It shows:
- resection of primitive tumor and of the liver metastases;
- repeated resections in the cases of liver recurrences;
- multimodal treatment with resection of the primary tumor and arterial percutaneous chemoembolization, Seldinger's technique, of the liver metastases associated in selected cases to percutaneous alcoholization of the same lesions.

Length of the video: 12 minutes.

### V051

DUODENUM-PRESERVING TOTAL RESECTION OF THE HEAD OF THE PANCREAS WITH PRESERVATION OF THE BILE DUCT AND BOTH ARTERIAL ARCADES FOR LOW GRADE MALIGNANT CYSTIC TUMOR

Shuichi Miyakawa, Akihiko Horiguchi, Tunekazu Hanai, Makoto Hayakawa, Shin Ishihara, Naotatu Niwamoto, Tadashi Satoh, Yuji Iwase, Haruo Yamamoto, Kaoru Miura
Dept. of Surgery, Fujita Health University, Toyoake, Japan

The duodenum-preserving resection of the head of the pancreas is an organ-saving procedure in the treatment of svere chronic pancreatitis. However, there are some problems, when the Beger's procedure is indicated to the patients with benign or low grade malignant tumor. First, tumor may remain in the paraduodenal pancreatic remnant. Second, the pancreatic fistula from the paraduodenal pancreatic remnant may be caused, because of remaining the normal gland of the pancreas, and the congenital obstruction of the accesory papilla. Third, the blood supply of the duodenum and the bile duct is disturbed, since the colaterals from the hepatoduodenal ligament and the retropancreatic space is not developped due to the normal gland. This conditions causes ishemia of the bile duct and the duodenum after the resection of the head of the pancreas, and results the early postoperative complications, such as perforation of the bile duct and the duodenum, and pancreatic fistula. For these patients, a new operative procedure is needed to prevent the forementioned problems. It is a total resection of the head of the pancreas, and the preservation of both blood supply and venous drainage of the bile duct and the duodenum. The metods of the preservation of the vessels during the total resection of the head of the pancreas is to preserve the SPDV and/or the IPDV during the exploration of the mesentericportal vein, the duodenal branches of the AIPDA and the ASPDA one by one taping between the vasa rectus, and dividing the pancreatic branches, and the pancreatic posterior menbrane. The Operative time was 429 minutes, and the mean blood loss was 810 ml. The mean postoperative hospitalization was 20 days. None of the patients had early or late postoperative complications. (Conclusion) The new operative procedure freed us from early postoperative complications. By Video, 13 minutes.

### V052

**CYSTPANCREATOJEJUNOSTOMY FOR ALCOHOLIC CHRONIC PANCREATITIS**

J.E.Monteiro da Cunha, M.C.C. Machado, T. Bacchella, S.Penteado, E.Abdo, J Jukemura, A Montagnini. H. W. Pinotti - Department of Gastroenterology, Surgical Division, São Paulo University Medical School. São Paulo, Brazil.

A 46 years old alcoholic male patient with calcifying chronic pancreatitis presented with marked abdominal pain due to pancreatic calcifications, duct dilatation and a pseudocyst at the tail of the pancreas. The video presents a Roux-en-y cystopancreatojejunostomy using the same jejunal loop for both the cyst and ductal anastomosis. Postoperative course was uneventful and the patient was discharged free of symptoms.

Twenty-five patients with chronic pancreatitis presenting with pancreatic cysts and duct dilatation were surgically treated at our institution with this procedure. Immediate pain relief was achieved in 96% of patients. pain recurrence was observed in 8.3% and was mostly related to alcohol abuse.

Conclusion: Pancreatic cysts in patients with chronic pancreatitis and duct dilatation may be successfully treated by means of cystopancreatojejunostomy using the same jejunal loop for the cyst and ductal anastomosis.

Lenght of the video: There are two copies one of 8 min (sound track in portuguese) and one of 15 min (sound track in english).

### V053

### USEFULNESS OF INVAGINATION FOR PANCREATOJEJUNAL ANASTOMOSIS IN PANCREATO-DUODENECTOMY

N. Morita, Y. Kobayashi, D. Hayashi, T.Inokuchi, K. Okamura, H. Shinagawa, T. Takahashi, T. Enoki, S. Noshima, K.Esato
First Deprtment of Surgery, Yamaguchi Univ, Ube., Japan

A 64 year-old female was admitted to our hospital for obstructive jaundice. Endoscopic retrograde cholangiogram demonstrated the obstruction of ampulla of Vater. Pylorus preserving pancreato-duodenectomy (PPPD) was performed for the cancer of ampulla of Vater. Our invagination method of pancreatojejunal anastomosis in PPPD will be displayed on video. Drainage tube was inserted in the main pancreatic duct after sharp dissection of pancreas. It was fixed with 3-0 Vicryl®. Pancreatojejunal anastomosis was done with two layer sutures with atraumatic needle Prolene® (3-0) threads. The first suture layer was attached the pancreatic parenchyma to the whole layer of the jejunum by interrupted suture. The second suture layer was attached the capsule of pancreas to the seromuscular layer of the jejunum, which was 2cm distant from the first suture line and that of pancreas was 1cm distant from the first line. The pancreatic duct drainage tube was pulled out from the jejunum after the suture of posterior layer. In a follow up study of this technique is easy and secure method for pancreatojejunal anastomosis, especially for a nondilatated pancreatic duct. The length of the video is 14 minites.

### V054

### PANCREATICODUODENECTOMY WITH PYLORIC PRESERVATION FOR CARCINOMA OF THE AMPULLA OF VATER (USING WATER JET DISSECTION).

Partensky C. Department of Digestive Surgery. Hôpital E. Herriot. 69437. Lyon. France.

A 59 year-old patient complained of jaundice due to a carcinoma of the ampulla of Vater. A pylorus preserving pancreaticoduodenectomy with pancreaticogastrostomy was performed.Water jet dissection was used for peripancreatic dissection, isolation of the portal vein and division of the pancreas. The Wirsung duct was isolated from the pancreatic parenchyma by selective dissection and divided at distance from the pancreatic surface. Anastomosis of the pancreatic remnant to the back wall of the stomach was done by running suture using transgastric external drainage of the Wirsung duct by a 6 French catheter. Rnning suture was also used for biliodigestive and duodenojejunal end-to-side anastomoses.

The patient underwent an uneventful postoperative course.

Transgastric external drainage of the Wirsung duct is used routinely in our practice when the Wirsung duct is not dilated and when the pancreatic parenchyma is not sclerotic.

VHS PAL. 12 mn.

### V055

### ISTHMIC PANCREATECTOMY AND HEPATIC CYTOREDUCTION FOR METASTATIC VIPOMA

C. PARTENSKY, Surgical Department, Edouard Herriot Hospital, Lyon, 69437, France.

A 55 year-old female patient was referred with severe secretory diarrhea and hypokalaemia. Fasting serum level of VIP was elevated. CT-scan and selective angiography demonstrated a primary tumor at the isthmus of the pancreas with hypervascular metastases to both lobes of the liver. Octreotide therapy proved to be ineffective.

A bilateral subcostal incision is perfomed. Multiple metastases are present at both lobes of the liver. Intraoperative ultrasonography demonstrates liver metastases, the largest of them being located in segment VII, and the primary tumor at the pancreatic isthmus.

Using ultrasonic dissection, a segmental isthmic pancreatectomy with pancreatogastrostomy is carried out. Technical details include parenchymal transsection at both sides of the pancreas using ultrasonic dissection, hemostasis with bipolar coagulation, selective isolation and transsection of the Wirsung duct, temporary intubation of the anastomosis by a transgastric catheter and periananastomotic application of fibrin glue.

Cytoreduction of the secundaries combines resection of multiple metastases at the right lobe and at segment IV with a left lateral segmentectomy.

Clinical and biological symptoms improved dramatically after operation. Fasting serum VIP returned to a normal level and Octreotid therapy was resumed.

Cytoreductive surgery is indicated for endocrine metastatic secreting tumors when the secretrory syndrome is uncotrollable by medical therapy.

Video VHS PAL 10 mn

### V056

### LITHIASIS OF THE DUCT OF SANTORINI IN PATIENT WITH PANCREAS DIVISUM

G. Romeo, F. Cardì, S. D'Antoni , D. Pluchino, L. Lombardo, G. Catania

Department of Surgery, Division of General and Oncological Surgery, University of Catania, Catania, Italy

Pancreas divisum is a congenital anomaly due to the failed fusion of the dorsal and ventral pancreas at the end of the eighth week of human gestation. When this anomaly occurs the duct of Santorini becomes the major ductal system of the exocrine pancreas, while the duct of Wirsung drains only the inferior head of the pancreas and the uncinate process.

The Authors report an extremely rare case of lithiasis of the duct of Santorini associated to pancreas divisum.

The patient complained of late postprandial colicky pain radiating to the back, accompained by vomit and weight loss. U.S examination showed marked dilatation of the main pancreatic duct (apparently the duct of Wirsung) and a calculus, 16 mm. of diameter, within its distal tract; gall bladder and biliary tract appeared normal.

ERCP shoewd a narrow, short duct of Wirsung that predicated the suspect of pancreas divisum, incannulation of the minor papilla, a few centimeters superomedial to the ampulla of Vater, showed a marked dilatation of the duct of Santorini and the presence of the stone. A CT-scan confirmed the dilatation of the duct of Santorini and the presence of the stone.

At laparotomy the dilated duct of Santorini, easily found by intraoperative echography, was opened longitudinally, the stone was removed and a latero-lateral pancreaticojejunostomy was performed on a Y-en-Roux loop. The patient, discharged in good health 10 days after the operation, was still asymptomatic at one year control.

The Authors discuss about the treatment of lithiasis of the duct of Santorini in association with pancreas divisum and conclude that in the case that they report pancreaticojejunostomy was the best choise.

### V057

### PANCREATICOGASTROSTOMY (PG) AFTER PANCREATODUODENECTOMY (DCP)

G. Romeo, G. Catania, F. Cardi, A. Iuppa, G. A. Petralia, L. Lombardo

Department of Surgery, Division of General and Oncological Surgery, University of Catania, Catania, Italy

In the past few years the use of PG for reconstruction after DCP has been reported more and more often. Several are the advantages of this technique: 1) gastric acidity and absence of enterokinase avoid pancreatic enzymatic activation, 2) no collection of pancreaticobiliary secretions and routine postoperative gastric decompression avoid anastomotical tension, 3) the anatomical proximity of the posterior gastric wall makes the anastomosis easy to accomplish technically without tension, 4) the excellent blood supply and the thikness of the gastric wall mean more secure anastomosis, 5) easy postoperative control of the anastomosis from the gastric side, 6) blind jejunal loop is avoided in case of obstruction of the pancreatic anastomosis. Aim of this video is to try to define, on the basis of our experience, the role this technique could take in reducing morbidity and mortality after DCP. From January 1993 to June 1995 nine pancreaticogastrostomy has been performed. Five patients had adenocarcinoma of the papilla, two had carcinoma of the distal coledocus and two had carcinoma of the head of the pancreas. Pancreaticoduodenectomy was performed with the pylorus-preserving modification. The neck of the pancreas was transected to the left side of the portal vein. The pancreatic duct was identified and individual bleeding vessels were ligated using single 4-0 polyglicolic suture. The stump of the remaining pancreas was freed from retroperitoneal attachment for 2 cm. The posterior wall of the stomach was approximated to the pancreatic stump without tension and the site of the gastric incision was easily found on the antrum. Pancreatogastrostomy was performed with two rows of interrupted suture, of which the external one always in non-absorbable suture. No tube was left through anastomosis but care was taken to avoid pancreatic duct obliteration. Biliary and digestive tracts were restored by hepaticojejunal and gastrojejunal anastomosis to the first jejunal loop. Two open drainage were always left in place near PG and choledocojejunostomy up to the sixth post-operative day; amylase level in the drainage fluid was assayed daily. A nasogastric tube was left in place until the return of intestinal activity. Octreotide was used prophylactically postoperatively.

In our series we had an operative death unrelated to PG; the major complications was a massive bleeding from the gastric side of the anastomosis occurred 3 days after the operation, associated to a partial deihescence of the anastomosis that we treated surgically.

Reported results after PG seems to demonstrate a sharp decline in the morbidity and mortality rates after DCP, but of course the real advantagese of this technique will be confirmed only after a greater clinical experience and, when possible, with randomised prospective studies.

### V058

### LAPAROSCOPIC ULTRASOUND FOR STAGING PANCREATIC MALIGNANCIES

R.Santambrogio, P.Bianchi, E.Opocher, M.Verga, A.Galli, F.Grasso and M.Montorsi. Departments of Surgery. Ospedale San Paolo, Ospedale Maggiore, University of Milan, Milano, Italy.

Notwithstanding the refinements in imaging techniques, a 44-59% of patients with pancreatic cancer proves to be unresectable at laparotomy. To this regard the role of laparoscopy for staging pancreatic malignancies has been greatly enhanced by the developements in laparoscopic equipment and techniques. Moreover, the recently available laparoscopic ultrasound (LUS) probes, carry potential advantages for staging purposes.
**Methods:** Laparoscopic examination was carried out through two to three 10-mm trocars inserted at the umbilicus, right flank and/or subxyphoid site. First step consisted in a thorough exploration of the peritoneal cavity, liver and other abdominal organs. A peritoneal lavage for citology was always carried out. Biopsy of any suspect lesions was performed when required. The second step consisted in LUS examination. LUS was performed using a 10-mm diameter linear-array ultrasound probe with a 7.5-MHz transducer. The examination started with LUS scanning of liver parenchyma to detect metastases; subsequently a LUS examination of pancreatic area was performed to define the site of pancreatic lesion and its relationship with vascular structures. Finally, a transgastric scanning of celiac trunk and of hepato-duodenal ligament was accomplished looking for malignant lymphadenopathies.
**Results:** LUS was performed in 13 patients with pancreatic cancer. Time required for a complete laparoscopic and LUS examination ranged from 25 to 45 minutes. No complications has been registered. LUS changed the planned surgical strategy in 38% of cases (5/13 patients). These changes were due to simple laparoscopic exploration in one patient and to LUS in the remaining four patients.
**Conclusions:** Laparoscopy with LUS seems to represent a useful examination for staging pancreatic malignancies prior to surgical resection.
Video length: 10 minutes.

### V059

### PPPD WITH PORTAL VEIN RESECTION AND EXTENSIVE RETROPERITONEAL NODE DISSECTION FOR PANCREATIC CARCINOMA

H.Shimada, K.Misuta, K.Kameda, I.Endo, A.Nakano
2nd Department of Surgery, Yokohama City University, Yokohama, Japan

PPPD was introduced for benign disease of the pancreas head or carcinoma of the ampulla of Vater, however in recent years it has been applied even for malignant neoplasms of the pancreas and the biliary duct.
During the last 5 years in our institution, PPPD was performed in 44 patients for malignant neoplasms. The operative morbidity rate was 59.1%, and mortality was zero, the postoperative weight loss was less than that of standard Whipple procedure. Of the 13 patients undergoing PPPD for pancreatic cancer, the median survival was 8.3 months and 1 year survival rate was 30.8% . We want to present our procedure of PPPD combined with portal vein resection, extensive retro-peritoneal node dissection and extrapancreatic neural plexus dissection for cancer of the pancreas head with stage II or III under the fourth edition of the UICC TNM classification.
(Surgical procedure) An upper midline incision was employed for laparotomy. The retroperitoneal dissection was performed around the abdominal aorta and IVC. The SMA root and celiac axis were isolated by dissecting of the neural plexus and the ganglion around the SMA, the celiac axis and the pancreas head. After division of the gastrocolic trunk, the middle colic v. and the first jejunal v. accompanied the inferior pancreatico duodenal v., the SMV was exposed just under the pancreas superiorly. The gastroepiploic artery was devided between first and the second branch, and the upper portion of duodenum was freed. The hepatic duct was divided after freeing of the gallbladder from the liver bed and the skeletonization in the hepato duodenal ligament was done leaving only the hepatic artery and the portal vein. And the portal vein was exposed dissecting lymphnodes around the common hepatic artery, the root of the splenic artery, and the retro pancreas head. Para-aortic and celiac axis lymphadenectomy and neurectomy became continious the retro peritoneal dissection which has undergone previously. After the pancreas was divided, the right side of neural plexus around the SMA was dissected.
At the Treitz's lig. the division of the jejunum and dissection of the left side neural plexus around the SMA, and the invaded portal vein was resected after clamping by vascular forceps.
The portal vein was reconstructed by vascular suture method using 5-0 prolene. Intraoperative radiation with 8 meV and 20 Gy was done around the root of the SMA. The reconstruction was employed Child's procedure, with end-to-side pancreaticojejunostomy by mucosal anastomosis, end-to-side hepaticojejunostomy and end-to-side duodenojejunostomy.

### V060

### LAPAROSCOPIC PALLIATION OF PANCREATICO-DUODENAL TUMORS

EM Targarona, Mi Pera, J Martínez, C Balagué, JJ Espert, S Pascual, M Trías.

Serv. de Cirugía. Hospital Clínic. Univ. de Barcelona. Barcelona.

Palliation of biliary or duodenal obstructions secondary to pancreaticoduodenal tumoral lesions is associated with a significant morbimortality. Surgical palliation is followed by an increased early morbidity, but nonsurgical options (percutaneous or endoscopic prostheses) are associated with a higher risk of cholangitis and obstruction, and need a replacement of the stent. Laparoscopic approach has been proposed as a feasible option, with lower incidence of early complications due its minimally invasive characteristics and a longer patent period in selected cases. **Aim:** To evaluate the early results of laparoscopic digestive biliary diversion in selected patients with irresectable pancreaticoduodenal tumors. **Material and methods:** Between set/93 and March/95 we have treated 5 patients with an irresectable pancreaticoduodenal tumor with a laparoscopic approach. **Results** Mean age was 70 y (50-87). 4 patients were diagnosed of pancreatic cancer and one of jaundice secondary to gastric neoplastic adenomegalies. Two patients received a cholecisto-jejunostomy alone, and in 2, a gastrojejunostomy was added. The fifth case was treated with a gastrojejunostomy, in a patient with duodenal obstruction with a prior wallstent cannulation of the bile duct. **Technique:** After an operative cholecystocholangiography that showed a patent cystic duct, a cholecysto jejunostomy was performed, using an endostapler. The anastomosis was completed with an intracorporeal running suture. Gastrojejunostomy was performed with a 65 mm endostapler, and the gastric and intestinal hole was closed with several aplication of the 35 mm endostapler. There were not major complications, but 1 catheter sepsis and 1 trocar site infection. No patient relapse jaundice during follow up. Mean stay was 10 days (7-17), and mean survival was 12 w (6-24). **Conclusion:** Laparoscopic approach should be considered as an useful alternative when considering palliative treatment of malignat jaundice, and comparative studies are needed to exactly know the potential role of this technique.

V061

### DUODENUM-PRESERVING TOTAL PANCREATIC HEAD RESECTION WITH PRESERVATION OF BILIARY TRACT FOLLOWING PANCREATIC DUCTAL ANASTOMOSIS

H. Yasuda, T. Takada, T. Uchida, T. Isaka, Y. Toyota
First Department of Surgery, Teikyo University School of Medicine, Tokyo, Japan

A complete resection of the head of the pancreas, with preservation of the duodenum and biliary tract, is presented by video. Previously reported techniques for duodenum-preserving pancreatic resection differ from our procedure. Previously other techniques accomplished only a partial resection, remaining a rim of pancreas along the duodenum. With our technique, duodenal blood flow is maintained, and no pancreatic parenchyma is left on the duodenal side.
Operative Procedure; After laparotomy, the posterior superior pancreatoduodenal artery is preserved by avoiding Kocher's maneuver. The superior mesenteric vein is exposed at the inferior border of the pancreas to performing a portal vein tunnelling procedure, and the pancreas is transected over the portal vein. The entire pancreatic head is resected from the cut edge towards the duodenum. The pancreas between the bile duct and the duodenum is isolated by elevating the head of the pancreas. The pancreatic duct is divided at its cofluence with the bile duct. The pancreatic ductal anastomosis is performed. To assure an adequate blood supply to the duodenum, retroperitoneal vessels must be preserved by avoiding Kocher's maneuver, and the posterior superior pancreatoduoduodenal artery must also be preserved.

### VIDEO PRESENTATIONS

### Topic: BILIARY

### V062

### TREATMENT FOR STENOSIS OF INTRAHEPATIC BILE DUCTS IN PATIENTS WITH CHOLEDOCHAL CYST

H. Ando, K. Kaneko, F. Ito, T. Seo, T. Ito, K. Ono, H. Takeuti

Department of Surgery, Branch Hospital, University of Nagoya School of Medicine, and Department of Surgery, Inazawa City Hospital, Nagoya, Japan

Excision of the dilated extrahepatic bile duct followed by biliary reconstruction has been recognizing as the treatment of choice for choledochal cyst. However, postoperative complications such as intrahepatic calculi developed even after the complete excision of a choledochal cyst. Since congenital stenosis of intrahepatic bile duct is more likely the cause of intrahepatic calculi, operative procedure for intrahepatic stenosis is reported.

Thirty-year-old female with choledochal cyst underwent an operation for stenosis of the intrahepatic bile duct. Stenosis excised around the perimeter at the opening of the common hepatic duct. Stenosis that involved the intraluminal membrane could be excised from the divided end of the common hepatic duct at the hepatic hilum. Postoperative follow-up demonstrated she had been in good health.

Stenosis of the intrahepatic bile duct should be treated at the initial operation for choledochal cyst. The need for a second operation or hepatic lobectomy may thus be avoided.

### V063

### CENTRAL HEPATIC RESECTION OF SEGMENTS I, IV & V FOR HILAR CARCINOMA UNDER LIVER OCCLUSION PRESERVING CAVAL FLOW.

J. Belghiti, R. Noun.

Department of Digestive Surgery, University Paris VII, Hospital Beaujon, Clichy, France.

A 43 year old female with carcinoma of the hepatic hilus underwent 6 weeks preoperative transhepatic biliary drainage. Arteriography demonstrated the patency of both arterial and portal branches. After improving jaundice a "en bloc" resection was performed including common bile duct, lymphadenectomy and segments I, IV and V. Resection was performed under partial liver exclusion including pedicle occlusion associated with venous occlusion of both middle and left hepatic veins with preservation of caval flow.

Central biliary resection resulted in 4 tumour free central bile ducts. For reconstruction a 50 cm jejunal segment was used with end-to-side cholangiojejunostomy.

No postoperative complication occurred and she was discharged from the hospital on the 17 postoperative days. She is free of recurrence two years later.

lenght of video : 12 minutes

### V064

### DIAGNOSIS OF CHOLEDOCHOLITHIASIS BY LAPAROSCOPIC ULTRASOUND DURING CHOLECYSTECTOMY

P.Bianchi, R.Santambrogio, E.Opocher, F. Ghelma, M.Verga, A.Galli, M.Panzera and M.Montorsi. Departments of Surgery. Ospedale San Paolo, University of Milan, Milano, Italy.

Since October 1993, we started an evaluation programme of patients with symptomatic cholelithiasis aiming to detect associated common bile duct (CBD) stones. Endoscopic retrograde cholangio-pancreatography (ERCP) was reserved for high-risk patients for CBD stones. Laparoscopic ultrasound (LU) during cholecystectomy was routinely performed to identify stones unsuspected preoperatively. One-hundred-sixty-four patients with symptomatic cholelithiasis were included into the study. 140 patients were at low-risk of choledocholithiasis, while 24 patients were at high risk: these patients were submitted to ERCP: in 12 cases CBD stones were found and removed by endoscopic sphyncterotomy. Mean time for LU examination was 8±2 min ranging from 5 to 15 minutes. In all patients the main intra and extrahepatic ducts were well documented, while in 9 cases (5%) the distal tract of the CBD was not well-visualized. In six patients (4%), unsuspected small stones into the CBD were found. A subsequent peroperative cholangiography confirmed the diagnosis. Transcystic duct approach was attempted in three cases and successful in two, while conversion to open laparotomy was performed in two cases. Two patients were successfully treated by postoperative endoscopic sphyncterotomy. No false positives were found. In two patients a small stone in the CBD was found during the follow-up period (two false negative): ERCP was performed in one case for biliary colic and in the other for mild acute pancreatitis. An endoscopic sphyncterotomy solved the problem. The sensitivity of the preoperative flow-chart was 56% and the specificity was 95%. The sensitivity of the preoperative flow-chart plus LU was 88% and the specificity was 95%. In conclusion, LU may be a real alternative to cholangiography during laparoscopic cholecystectomy. Some failures can be prevented by additional experience and the use of a LU probe with flexible tip.

Video length: 10 minutes

### V065

### BILIARY DUCT AND RIGHT VASCULAR PEDICLE LESIONS SECONDARY TO LAPAROSCOPIC CHOLECYSTECTOMY

E. de Santibañes, M. Ciardullo, J. Pekolj, J. Grondona, R. Sendin, J. Sívori.
HPB Surgery Section, General Surgery Service, Hospital Italiano, Buenos Aires, ARGENTINA

A video of the surgical repair of an important common bile duct and right vascular pedicle injuries is presented. It was performed in a woman of 58 years old, that was operated in other hospital with the diagnosis of simple cholelithiasis, with the video laparoscopic procedure. During that operation the surgeon confused the cystic duct with the common bile duct, so be cutted and then ligated it above the hepatic confluence (Bismuth IV). Further he didn't performed the intraoperative colangiography and did not advise the lesion, so he had continued the operation in a wrong way and ligated the right vascular pedicle (right hepatic artery and right portal branch). The video details the biliary duct injury approach, the hepatic confluence dissection, the use of the intraoperative ultrasound, the section of the hepatic parenquima with the cavitron ultrasonic aspirator (CUSA) and the use of argon beam and synthetic glues for hemostasia. Further the double hepatoyeyunostomy with a Roux-en-y loop in one layer with interrupted stitches with polypropilene 7/0 is showed.Transanastomotic and transhepatic catheters were placed and a feeding jejunostomy (Witzel procedure) was performed. We conclude that this technique is the best choice for the repair of these serious bile duct injuries and during ever laparoscopic cholecystectomy the intraoperative cholangiography must be carried for advise the session and convert in open surgery for the immediate intent of repair.

(The video lasts 12 minutes)

### V066

### FIBROMA OF THE HEPATIC HILUM

S. Duca, C. Iancu, O. Bălă
Department of Surgery, Surgical Clinic III, University of Medicine Cluj-Napoca, Romania

We report the case of a 54-year-old female patient who was admitted for right upper abdominal pain. Imagistic studies(CT scan, i.v. cholangiography) excluded gallbladder stones but revealed a well defined tumoral mass in the hepatic hilum. A laparoscopic approach was attempted. Laparoscopic cholecystectomy was performed in order to gain access to the tumoral mass. The tumoral mass was then enucleated from behind the common bile duct and extracted. Its dimensions were 35/25 mm. Pathology revealed the structure of a fibroma. The patient is simptom free at 18 month follow up. We consider this case interesting due to its rarity and due to the laparoscopic approach of the tumor.
The length of the videotape is 9 minutes

### V067

### TREATMENT OF CHOLECYSTO-DUODENAL FISTULA DURING LAPAROSCOPIC CHOLECYSTECTOMY (VLC). (video 12 min.).

A.Faggioni, A.Mandrini, G.Moretti, P.Viazzi,A.Noceti.

Department of Surgery, Ospedale Genova-Nervi, Genoa-Italy.

Aa. present videolaparoscopic treatment of cholecysto-duodenal fistulas during VLC. Between October 1991 and October 1995, out of a total of 814 biliary operations (747 VLC, 63 VLC with choledocolithotomy, 4 VLC with choledoco-duodenostomy) we encountered 5 cholecysto-duodenal fistulas. Only in 1 case the diagnosis was pre-operatively suspected, owing to the presence of pneumobilia indicated by US scans. In 4 cases the treatment was laparoscopically performed; in 1 case the procedure was converted to open surgery on encountering an empyema with strong peri-cholecystic adhesions. In 3 cases the procedure consisted of isolation of cholecysto-duodenal fistula and laparoscopic mechanical resection/suture; in 1 case, owing to the presence of a thin hole in the duodenum, after detaching the gallbladder, we performed a suture with separated absorbable stitches. After the cholecystectomy a sub- hepatic drainage tube was inserted and then removed on post-operative day 2 or 3. The patients were released between post-operative day 6 and 10. Follow-up of the patients is normal 32, 14, 8 and 1 months after surgery; in particular radiographic and endoscopic examination of the duodenum did not show morphologic or functional pyloro-duodenal alterations.

### V068

### LAPAROSCOPIC CHOLEDOCOLITHOTOMY IN PATIENTS PREVIOUSLY SUBJECTED TO CHOLECYSTECTOMY AND ENDOSCOPIC PAPILLOTOMY (EPT). (video 12 min.).

A.Faggioni, G.Moretti, A.Mandrini, A.Noceti,P.Viazzi.

Department of Surgery, Ospedale Genova-Nervi,Genoa, Italy.

Aa. present 3 cases of common bile duct lithiasis occurred 1, 13, 20 years after cholecystectomy. The patients underwent EPT but, because of the failure to remove the stones, a videolaparoscopic treatment was performed. The first patient (72 year old male) underwent laparoscopic cholecystectomy 1 year before. After the failure of endoscopic retrograde extraction of common bile duct stones, a videolaparoscopic lithotomy (2 stones) was trans-choledocally performed. The procedure involved the use of fiberscope, Dormia basket and Fogarty balloon. A trans- choledochic T tube was inserted and repair of the bile duct was completed with separated absorbable stitches. The patient was released on post-operative (p.o.) day 7 and the T tube was removed on p.o. day 19. The second patient (61 year old female) underwent cholecystectomy 20 years before via a right trans-rectal scarring. After EPT and double failure of endoscopic stones extraction, the patient was laparoscopically treated: viscerolysis and fibercholangioscopy with trans-cystic lithotomy (2 stones) were performed; the cystic duct was closed with loops. The patient was released on p.o. day 4. The third patient (80 year old female) underwent cholecystectomy 13 years before via a median xipho-umbilical scarring. After EPT and double failure of stones extraction, a videolaparoscopic lithotomy was performed (4 stones) with the use of forceps and a trans- choledochic approach. Repair of bile duct was completed with separated absorbable stitches and the patient was released on p.o. day 6. Follow-up of the patients is normal 12, 6 and 1 months after surgery. It follows from our experience that a videolaparoscopic approach is indicated before laparotomy, if endoscopic retrograde treatment is not resolutive in patients with common bile duct stones, previously subjected to cholecystectomy.

### V069

### EXPERIENCE WITH PERCUTANEOUS CHOLANGIOSCOPY (PC).

F.Fiocca, FM Salvatori, E Grasso, M Bezzi, G Scopelliti M Cristaldi, P Ricci, D Apa, P Rossi, V Speranza.
II Surgical Clinic and III Dept of Radiology - University "La Sapienza" - Rome, Italy.

97 pts underwent PC with a 3.9 or 5.0 mm endoscope: 85 2-7 days after a transhepatic fistula dilated up to 12 or 16 F, 12 after removal of a surgical implanted T-tube. 46 pts with biliary stones were all cleared after 1-4 treatments (mean 1.8), in 12 by means of Electrohydraulic lithotripsy, in the others with fluid flushing. 5 pts had recurrent stones and they were retreated.
21 biopsies, out of 41, confirmed a malignat stricture. Surgical stitches were removed in 8 pts. There were 4 massive bleeding that required embolization and 2 perforations of the bile duct repaired with stenting. No mortality was observed. Minor complications were 18 pts with nausea and intollerance (6 fluid overload) and 15 cases of minor bleeding. We conclude that PC is a safe treatment for biliary calculi, useful alternative to surgery and for diagnosis and treatment of biliary diseases, especially strictures.
This procedure should be done in strictly cooperation with an experienced radiologist, endoscopist and surgeon.
The video will last 10 minutes.

### V070

### LYMPH NODE DISSECTION FOR GALLBLADDER CANCER BY LYMPH NODE STAINING METHOD

H. Isozaki, K. Okajima, S. Morita, H. Hara
Department. of Surgery, Osaka Medical College, Osaka, Japan

Knowledge of the lymphatic flow from the gallbladder is indispensable for rational lymph node dissection of gallbladder cancer. Fine activated carbon particles (CH40) injected in the tissue (especially when injected in lymph node) are taken into the lymphatics immediately, and the lymphatic vessels and lymph node can be visualized by staining black. By this method, regional lymph nodes relating to gallbladder can be removed more easily. The lymph node dissection technique using CH 40 is shown in this video.

**(THE PATIENT)**

A 68 year old female with carcinoma of the gallbladder associated with pancreatobiliary maljunction.

**( OPERATIVE PROCEDURE)**

Before lymph node dissection, 1ml of CH40 was injected slowly into the paracholedochal lymph node (No.12b) which is the first lymph node station of gallbladder. Then, many lymph nodes which receive a lymphatic flow from paracholedochal node were stained in black, indicating the extent of rational lymph node dissection. The following stained lymph node were dissected : lymph node in the hepatoduodenal ligament (No. 12), behind pancreas head (No13a), along the common hepatic artery and celiac trunk (No. 8a,8p,9), at the root of mesentery (No. 14a, 14d), paraaortic lymph nodes (No.16). The dissection of paraaortic lymph nodes stained in black is thought to be important for curability of gallbladder cancer.

**(RESULT)**

The patient is alive three years after the operation.

**(CONCLUSION)**

Lymph node staining method was useful for the radical lymph node dissection of gallbladder cancer.

The length of video; 10 minutes.

### V071

### ENDOSCOPIC TREATMENT OF CHOLEDOCHOLITHIASIS

Karcz D., Zajac A., Szura M.

*Ist Department of General and GI Surgery, Jagiellonian University, Kraków, Poland.*

Endoscopic treatment of choledocholithiasis is becoming the most effective method.The authors are presenting own experience with endoscopic treatment of choledocholithiasis in 978 cases with choledocholithiasis before and after cholecystectomy. In each case bile stones removal was preceded by endoscopic papillotomy. Large concrements were crushed before their removal using mechanical, hydraulic, or ultrasound methods Moreover transduodenal visualizing of bile ducts lumen enabled detection and removal of intrahepatic bile ducts stones which is impossible using radiological methods.. The effectiveness of the applied method in the treatment of choledocholithiasis was 95%, and the rate of complications was 2.8%. High effectiveness of the endoscopic treatment of choledocholithiasis as well as low complication rates make us recommend this procedure as a method of choice in cases of choledocholithiasis.

### V072

### POST LAPAROSCOPIC CHOLECYSTECTOMY STRICTURE : HEPP-COUINAUD PROCEDURE WITH POSTERIOR INTRAHEPATIC GLISSONIAN APPROACH

B. Launois, J. Terblanche
Department of Digestive Surgery and Transplant Unit, CHR Pontchaillou, Rue Henri Le Guilloux, 35033 Rennes, France

A new technique which has simplified the biliary tract repair is described. The posterior intrahepatic glissonian approach brings the sheaths to a more superficial position, in essence making them superficial. Therefore the Hepp-Couinaud procedure is easy to perform with a long anastomosis on the left hepatic duct.

### V073

### EXTEDED RIGHT LOBECTOMY WITH CAUDATE LOBECTOMY AND COMBINED PORTAL VEIN RESECTION FOR HILAR CHOLANGIOCARCINOMA

SG. Lee, SK. Kim, YJ. Lee, KM. Park, PC. Min
Department of Surgery, Asan Medical Center, Seoul, Korea

A 59-year-old women presenting with deep jaundice and fever underwent bilateral PTBD to relieve Jaundice and cholangitis.

Detailed cholangiogram through PTBD revealed Bismuth type III hilar bile duct carcinoma and hepatic angiography showed tumor encasement of hight anterior hepatic artery and caudate branch of left portal vein.

Serum total bilirubin was 3.1 mg/dl and high fever persisted just before operation. After exploration, right hepatic artery and caudate branch of portal vein were invaded by tumor. Extended right lobectomy including caudate lobe and bile duct resection and R2 lymph node dissection was performed with wedge resection of portal vein.

Four hepatic ducts were exposed for reconstruction. Postoperative course was uneventful.

Preoperative CA 19-9 level was above 200 u/ml and it dropped below 10 u/ml at 30th day after resection. This video shows technical details of extended right hepatectomy and caudate lobectomy for advanced hilar cholangiocarcinoma.

### V074

Endolaparoscopic Approach for Recurrent Pyogenic Cholangitis

Michael K. W. Li, W. T. Siu, H. T. Leong
Department of Surgery, Pamela Youde Nethersole Eastern Hospital

Recurrent Pyogenic Cholangitis is a common condition seen in the Far East. Patient can present with life threatening cholangitis and emergency laparotomy carries a significant morbidity and mortality. The use of emergency ERCP to drain the obstructed system and broad spectrum antibiotcs had reduced significantly the need for emergency operations. Majority of these patients will need a definitive drainage procedure such as choledochodoudenostomy or choledochojejunostomy when the acute situation settled. This 10 minutes video will demonstrate our technique of Endolaparoscopic approach to this condition in this era of minimal access surgery. Three patients had received this treatment so far. All had dilated common and intrahepatic ducts with severe cholangitis on admission. Emergency ERCP were performed with temporary drainage using either nasobiliary catheters or stents. Laparoscopic common duct explorations were carried out to clear the ductal system of sludge and stones. This was followed by cholecystectomy and choledochoduodenostomy using a one layer side to side anastomosis. The average operating time was 180 minutes. Postoperative recovery was uneventful. In conclusion, we are encouraged with our initial experience and feel that there is a place for this approach in selected patients with this condition.

### V075

**LAPAROSCOPIC CHOLECYSTECTOMY IN ACUTE CHOLECYSTITIS COULD BE A SAFE PROCEDURE ?**

***Medhat M. Sabet, Sarwat M. Ali, El-Tabei H. and Essam A. Shelbaya.***

**Minia University Hospital**

**The past years have been a time great excitement in laparoscopic surgery and laparoscopic cholecystectomy in particular. Sixty nine patients suffering from acute calcular cholecystitis subjects in laparoscopic procedure were included in this study. Patients were collected at random, 41 patients were women and 28 were men, patients aged 26 - 84 years with a mean age 58.5 years, laparoscopic cholecystectomy could be done in 54 cases (78.26%). Cholecystectomy was conducted safely in 42 cases (60.87%). In 18 cases laparoscopic cholangiography through cannulation of gall bladder or cystic duct was induced and it was successful in 14 cases and laparoscopic cholcystectomy was proceeded in 12 cases (17.39%). Minor complications occured in 9 cases and included bile leakage in 4 cases, umbilical sepsis in 2 cases and 3 cases of abdominal wall haematoma. Major complication in the form of duodenal perforation occured in one case. In fifteen patients (21.73%) the operation were converted into open procedures because of difficult or unclear anatomy in 8 cases, failed cholangiography in 4 cases, cholangiographic evidences of common bile duct stones in 2 cases, cystic artery bleeding in one case and perforated duodenum in one case. No mortality occured in this group of patients. With adequate experience, proper judgement at selection of cases of acute cholecystitis suitable for laparoscopic cholecystectomy and with the help of operative laparoscopic cholagiography and endoscopic sphincterotomy, laparoscopic cholecystectomy could be a safe procedure.**

### V076

**Acute Cholecystitis and Empyema : Laparoscopic U.S. Open Cholecystectomy**

***F.A. Mekky (M.D)***

Comparative study of 2 groups of patients was done to compare the result of laparoscopic (LC) versus open cholecystectomy (OC) in patients with acute cholecystitis and empyema of the gallbladder.

LC group included 20 patients with clinical diagnosis of acute cholecystitis, abdominal ultrasound with stones in GB or impacted in the cystic duct.

As well as over distended gallbladder with infected bile or pus. Histological examination with evidence of acute inflammation from June 1991 to Ma 1994.

O"C group included 45 patients with the same characteristics operated upon in the same period.

Regarding the results, it was found that the mean length of the operative procedure was 75 minutes in LC and 110 minutes in OC. The mean postoperative stay was 3.2 days in LC and 12 days in OC.

LC appears to be safe and beneficial option in the management of acute cholecystitis and empyema of GB as compared to OC.

### V077

LEFT HEPATECTOMY FOR BILIOSTASIS AFTER A LAPAROSCOPIC BILIARY INJURY.

M.Morino, C.Garrone, V.Festa, C.Miglietta.
Istituto di Clinica Chirurgica I, University of Turin.

The video presents the case of a 28 years-old female who underwent laparoscopic cholecystectomy (LC) for symptomatic gallbladder lithiasis at another Institution.

The immediate postoperative course was uneventful and the patient was discharged. At the 30th postoperative day she was readmitted for a surgical drainage of a subhepatic biliary collection. After this second operation the patient developed a biliary fistula. At this time she was referred to our Institution. An endoscopic retrograde cholangiopancreatography (ERCP) showed no leakage, no fistula, normal positioning of laparoscopic clips but failed to opacify segments IV and III. The injection of contrast through the subhepatic drainage under fluoroscopy combined with a cholangio-CT-scan showed an intrahepatic transection of segmental bile ducts III and IV.

The video shows the laparoscopic procedure, the diagnostic work-up and the left hepatectomy that we were obliged to perform in order to resolve biliostasis. In fact the two transected biliary segmental ducts were too small to allow a safe biliary reconstruction. The postoperative course was uneventful and the patient was discharged on the 8th postoperative day.

At the follow-up she remains asymptomatic 4 months after the last procedure.

Length of the video: 15 minutes.

### V078

### RIGHT HEPATIC TRISEGMENTECTOMY WITH TOTAL CAUDATE LOBECTOMY AND EXTRAHEPATIC BILE DUCT RESECTION AFTER PORTAL VEIN EMBOLIZATION FOR ADVANCED HILAR CHOLANGIOCARCINOMA

Y. Nimura, J. Kamiya, S. Kondo, M. Nagino, E. Sakamoto, Y. Goto
The First Department of Surgery, Nagoya University School of Medicine, Nagoya, Japan

We will demonstrate preoperative portal vein embolization of the right and left medial segmental branches and anatomical liver resection employing a right hepatic trisegmentectomy with total caudate lobectomy and extrahepatic bile duct resection for advanced hilar cholangiocarcinoma.
A 61 year-old male patient complained of jaundice. Selective cholangiography through the transhepatic biliary drainage catheters revealed diffuse cancer extension predominantly in the right hepatic duct and left medial segmental duct and proximal extension up to the confluence of S2 and S3 ducts. From these findings, right trisegment portal vein embolization was performed and the volume of the left lateral segment changed from 18% to 22 % of the whole liver. The operation was performed on September 20, 1994, 19 days after the portal vein embolization.
After dividing the right and middle hepatic arteries, the right portal vein was resected, and caudate lobe branches and left medial segmental branches of the vein were entirely divided. Then the umbilical portion of the left portal vein was completely detached from the umbilical plate. Then the right lobe was mobilized and the entire short hepatic veins and the right hepatic vein were divided and closed.
Next, the liver transection was carried out along the umbilical fissure on the left of the falciform ligament, and the caudate lobe was resected en bloc together with the right trisegment of the liver. The left lateral segmental ducts were resected proximal to the confluence of S2 and S3 ducts with free margins and reconstructed with a jejunal loop.
Postoperative course was uneventful except for hyperbilirubinemia. The patient has been enjoying a high quality of life and surviving without tumor recurrence for 1 year and 3 months.

### V079

### BILIARY CONFLUENCE EXCISION WITH EXTENDED RIGHT HEPATECTOMY + TOTAL CAUDATE LOBECTOMY FOR HILAR CHOLANGIOCARCINOMA

G. Nuzzo, F. Giuliante
Geriatric Surgery Unit, Catholic University, Rome, Italy.

The resection of the caudate lobe is mandatory for hilar cholangiocarcinoma Types II and III according to Bismuth-Corlette classification and to Gazzaniga classification. The video shows the case of a 67 year old male with a hilar cancer classified as Type IIIa according to Bismuth-Corlette classification and as Type II according to Gazzaniga classification. The patient presented with deep jaundice, lasting by 3 weeks. Abdominal US revealed enlarged intrahepatic bile ducts and CT scan documented a 3.5 cm mass at the biliary confluence. ERCP demonstrated a stenosis of the main biliary confluence and two endoscopic stents were placed to drain the right and the left bile ducts. Angiography did not document vascular neoplastic infiltration. Two weeks later, at surgery the involvement of the right vascular structures of hepatic hilus was found and the neoplastic diffusion along the right bile duct was observed. Complete lymphoadenectomy of hepatic pedicle was performed and the right hepatic artery and the right portal branch were transected. The vena cava was skeletonized from right to left and from below to above and the venous branches from the segment I were progressively transected up to confluence between suprahepatic veins and vena cava. Parenchimal transection was performed using a kelly clamp and biliary confluence excision with extended right hepatectomy and total caudate lobectomy was performed. In order to avoid any venous stasis to the defunctionalized jejunal loop, during hepatic resection no parenchimal ischemic manoeuvre was carried out. Intraoperative histologic examination of the proximal section margin of left biliary duct stump on the resected specimen, excluded neoplastic infiltration. Biliary continuity was restored by a Roux-en-Y left hepatico-jejunostomy, with a 70-cm jejunal loop. Histology revealed a G2 infiltrating cholangiocarcinoma. The patient developed an external biliary fistula which closed spontaneously after 21 days.

### V080

### LAPAROSCOPIC CYSTOGASTROSTOMY FOR CHRONIC PSEUDOCYST OF PANCREAS - TOTALLY INTRACORPOREAL HAND SUTURED.

C. PALANIVELU *M S Mch.*, P S Rajan, S V Sivakumar, K Sendhilkumar, R Parthasarathi, Dept. of Surgical Gastroenterology, Coimbatore Medical College and Hospital, INDIA.

Widespread success of laparoscopic cholecystectomy has given a way for advanced laparoscopic surgery. Cystogastrostomy for chronic pancreatic pseudocyst is being successfully carried out by laparoscopic method.

Since January 1994, 7 patients with chronic pancreatic pseudocyst were treated successfully by completely intracorporeal hand sutured cystogastrostomy. Age ranging between 22 and 56 years, 4 males and 3 females. One had pancreatic abscess during the acute phase - had external drainage - had recurrent pseudocyst after 5 months - treated by laparoscopic method.

**Procedure :** Adhesiolysis, anterior gastrotomy, everting stitches of the anterior gastric wall, confirmation of the cyst by percutaneous aspiration, stay stitch on the summit, circumferential excision of the posterior wall of stomach and cyst wall of 3 cms in diameter, emptying of the cyst contents, continuous or interrupted vicryl stitches to the cut margin for haemostasis and suturing of the anterior gastrotomy was the standard procedure performed in all the cases.

**Conclusion :** Chronic pseudocyst pancreas can be effectively and safely managed by laparoscopic method.

### V081

### LAPAROSCOPIC SPLENECTOMY - RETROGASTRIC ANTERIOR APPROACH FOR LARGE SPLEENS

C. PALANIVELU *M S Mch.*, P S Rajan, S V Sivakumar, K Sendhilkumar, R Parthasarathi, Dept. of Surgical Gastroenterology, Coimbatore Medical College and Hospital, INDIA.

Initial success of laparoscopic splenectomy for normal sized and slightly enlarged spleens has extended its indication to large sized spleens.

Since 1993, 5 cases of large sized spleens over 20 cms were successfully treated by laparoscopic approach. Age ranging between 24-56 years. 3 were male and 2 were female. Five ports were used in all the cases and semilateral position was adopted in all cases.

**Procedure :** Spleen was mobilised in the following steps : 1) Splenic flexure mobilisation. 2) Gastrosplenic ligament was divided after clipping the short gastric vessels. 3) Ligation and division of splenic artery and vein. 4) Division of lateral peritoneal fold.

**Spleen removal :** Spleen was removed through minilaparotomy. Cholecystectomy was combined in two cases. Initial ligation of splenic artery makes blood loss to minimum. Patients were discharged between 3 to 5 days.

**CONCLUSION :** Large sized spleen (20 cms) can be safely mobilised laparoscopically and extracted through minilaparotomy.

### V082

**Laparoscopic Transcystic Mechanical Lithotripsy (LTML) of bile duct stone.**

J.Pekolj, S Eubanks, I Mc. Lean.,E. de Santibañes, J.Sívori.

HPB Surgery Section, General Surgery Service, Hospital Italiano, Buenos Aires ARGENTINA.

The size of the bile stones more than 6 or 8 mms, is for some authors the point were the laparoscopic transcystic approach has to be changed to the laparoscopic choledochotomy. These dimensions are related to the size of bigest balloons employed for the dilatation of the cystic duct. However the posibility to perform lithotripsy inside the bile ducts, could change this principle. The technique of LTML is similar to employed for the gastroenterologist in the endoscopic approach, or for us in the percutaneous access. We use a endoscopic 4 multifilament wire soft Dormia basket with 2 x 4 open size. The total length is 220 cm and 7 French size when the basket is closed. It has a lateral way for injection of radio opaque dye. For crush the stone, we introduce a straight metallic canula in one of the lateral trocars.The basket comes in trougth this canula.The cystic duct is opened close to junction with common bile duct. Once the stone is catched by the basket, both are pulled back togheter near to the cystic duct. The metallic canula comes in the duct, and again the basket is pulled back against the canula, till the stone is broken. The result are many pieces of stones in the CBD. It is cleaned combining extraction with Dormia Basket, and flushing bile duct with saline solution; previous IV administration of 1mg. of Glucagon.In this video we show first an " in vitro" procedure, and late an "in vivo" procedure, of a case with a stone of 3 X 2 cms size. The combined radioscopic and laparoscopic control make the procedure safer and easy to learn.In a serie of 94 cases of bile duct stones treated by laparoscopic approach, we perform LTML in 7 ( 7,4%) patients.

**Conclusion:** With a similar technique described by the gastroenterologist, it is possible to perform LTML of bile duct stones.The design of flexible metallic canulas make safer the lithotripsy. This procedure increase the indications of the transcystic approach, avoiding in the successful cases the laparoscopic or open choledochotomy.

**(The tape last 9 minutes)**

### V083

**Video laparoscopic transcystic choledocholithotomy**

J.Pekolj, O Mazza, E de Santibañes, J. Sívori.
HPB Surgery Section, General Surgery Service,
Hospital Italiano, Buenos Aires, ARGENTINA

In the era of laparoscopic cholecystectomy (LC), the treatment of bile duct stones associated to gallstones is still no definitively resolved, however the laparoscopic transcystic choledocholithotomy (LTC),is the technique most accepted by the surgeons.In this video, different cases are presented, to describe the most common steps: intraoperative cholangiogram, milking of the cystic duct, dilatation of the duct with balloon, flushing of bile ducts, extraction of the stones with Dormia basket, and the control with fibrocholedochoscope. The main case, was performed under radioscopic guide, with a soft endoscopic Dormia basket. The advantages of this basket of 7 French size, are: **1.** To be soft, make it safer to work, avoiding the perforation of the walls of the duct. **2.** It has a lateral way for injection of radio opaque dye, making easier the control under radioscopy. **3.** The size of the open basket is 2 x 4 cm, making possible to catch almost all the stones. **4.** The total length is 220 cm., and the nurse how has on charge the injection of the dye and to open and close the basket, works far and confortable .**5** The basket is the ideal to perform mechanical lithotripsy. In the same case, we show how resolve proximal stones to the cystic duct, using " external manoeuvres" on the proximal bile duct, and with the "luxation" of the cystic duct. This duct was closed with a Endoloop.In our Service, we perform sistematically intraoperative cholangiogram, and we resolve the bile duct stones in the same operation. We indicated the LTC in 87 cases, among 1231 patients with LC ( 8 %). In the last 64 consecutives cases, the aplicability was 85 %, and the effectiveness of 93%. In unsuccessful cases, we performed open surgery, or laparoscopic choledochotomy with primary closure of the common bile duct.

**Conclusion:** The LTC is an high effective treatment for bile duct stones asociated to gallstones.The training in the different manoeuvres, and the availability of instruments as guide wires, angioplastic balloons, baskets and scopes, increase its application and effectiveness .

**(The tape last 14 minutes)**

### V084

**Simultaneous percutaneous treatment of acute cholecystitis and subfrenic abscess**

J.Pekolj, L.Mc Cormack, de Santibañes E., SívoriJ.,
Section HBP Surgery, General Surgery Service, Hospital Italiano, Buenos Aires ARGENTINA

The percutaneous cholecystostomy (PC) is a useful procedure for the treatment of acute cholecystitis in high risk patients. Also the percutaneous drainage of abdominal abscesses has a high effectiveness. However there are not many comunications combining both procedures in the same patient. Some authors described as a contraindication for PC, a fluid collection asociated to acute cholecystitis

In this tape, we present a 72 years old high risk patient with acute acalculous cholecystitis and a subfrenic abscess associated.The PC was performed with local anestesia with combined radioscopic and ultrasonic guide.Whith a Seldinger technique, a 8.3 pigtail nephrostomy catheter was placed in the gallbladder. The injection of radio opaque dye by the catheter, showed a perforation in the fundus of gallbladder.

The subfrenic abscess was drained with the same technique than PC, and a 12 French pigtail catheter was placed, and 120 cc of bilious purulent fluid were obtained during the procedure. The patient improve the general status and didnt develop complications related to the procedure. The subfrenic catheter was retired on day 7, and the patient was out of hospital on day 8. The PC was closed the day 10, and was retired day 26.

In our serie of 61 PC, only in two cases was necessary to add to gallbladder drainage, a catheter in associated fluid collection.

**Conclusion:**The PC is effective to resolve acute cholecystitis included the advanced cases with associated fluid collections. A close follow up is necessary, and in the cases were the patient's status doesn't improve, the open resolution must be considered.

**(The tape last 10 minutes)**

### V085

**Videofibroendoscopic evaluation of biliary tree.**

J.Pekolj, I.Mc Lean, J.Sívori, M. Ciardullo, E. de Santibañes
HBP Surgery Section, General Surgery Service, Hospital Italiano, Buenos Aires ARGENTINA.

The endoscopic evaluation of biliary tree ("choledochoscopy") is an effective method for evaluation of bile stones and tumors. However before the "laparoscopic era" few surgeons use routinly the scopes in the biliary procedures. The development of small video cameras, and the training with other endoscopic devices, make nowadays easier the use of this instrument by the surgeons. In this tape we describe a fibrocholedochoscope with an external diameter of 4.5 mm (15French), the instruments as Dormia Basket, Fogarty balloon, biopsy forceps, and electrode for electrohidraulic lithotripsy (EHL).We present the four accesses were we have experience: **1. Transhepatic approach:** a patient with liver transplantation and a big stone in common hepatic duct above the biliary anastomosis, was resolved by this access. Under endoscopic guide, EHL was performed. The endoscopic study showed the cystic duct of the donor and recipient, and the sutures of biliary anastomosis. The ampulla was free and the fibroscope past to duodenum. The left biliary tree was normal. **2. Laparoscopic transcystic approach:** we use it selectively and without an extra port for the fibroscope. In a case of multiple stones resolved by transcystic access under radioscopic guide; the study was performed to discard residual stones. It was possible to explore the distal and the proximal biliary tree. No stones were found, the ampulla was free, and a Fogarty catheter past to duodenum.**3. Open choledochotomy approach:** It was used to discard intraoperative residual stones, and to confirm the presence of a distal tumor with mucosal invasion of biliary tree.**4. Transfistular approach:** The procedure was performed by the tract of a T tube, to explore the biliary tree in a case of residual stone.

**Conclusion:** The videofibroendoscopy is an excellent procedure for the evaluation and to assist the treatment in bile ducts. The training for the surgeons is nowadays easier, and we promote a more frecuent use of the fibroscope, during biliary procedures. The selection of the type of the scope, will be related to the accesses to be employed.

**(The tape last 11 minutes).**

### V086

### Laparoscopic primary closure of the common bile duct

J.Pekolj, R. Sendin, I. McLean, J.Sivori, E. de Santibañes
HBP Surgery Section, General Surgery Service, Hospital Italiano, Buenos Aires ARGENTINA.

Biliary drainage following exploration of the common bile duct is still a subject of controversy. The development of fibroscopes, the improvement of the sutures and the techniques for closure of biliary tree, and the laproscopic approach are increased the interest for primary closure of the bile duct (PCB). For a safe procedure, W. Mayo and P. Mirizzi stresseed four strict requirements: 1.Patency of Ampulla of Vater, 2.Complete removal of all intraductal calculi, 3.Presence of normal pancreas; and 4.Meticulous suture of the duct. The first two conditions are confirmed with the use of a fibrocholedochoscope. The third with the selection of the patients and evaluation during surgery, and the fourth is resolved with training. In the last two years, we performed eleven open and five laparoscopic PCB.

In the tape we present a case of bile duct stones, were the transcystic approach was not possible, and it was resolved by laparoscopic choledochotomy, extraction of stones and primary closure .The biliary tree was evaluated proximaly and distally, the ampulla was open and was possible to pas a Fogarty ballon to duodenum. No residual stones were found. The pancreas was normal. The closure was performed with interrupted stitches of Maxon 4/0, with intracorporeal knots. No biliary drainage was used. A subhepatic sump drainage was left in place for 48 hours. The patient left the Hospital in his third postoperative day. No patients in our serie of PCB developed bile leaks, coleperitoneum, intraabdominal biliary collections, jaundice, residual stones or other postoperative complications.

**Conclusion:** The laparoscopic PCB in patients with normal intraoperative endoscopic evaluation of biliary tree, normal pancreas, and performed by surgeons with training in laparoscopic sutures; is a effective procedure for treatment of bile duct stones when the transcystic approach is unneffective or non aplicable. The ausence of biliary drainage, makes the postoperative course closer to laparoscopic cholecystectomy, than to laparoscopic choledochotomy with T- tube.

**(The tape last 10 minutes)**

### V087

### The difference between the laparotomic and the laparoscopic cholecystectomy.

D.Pick,M.D., R. Kolvenbach,M.D.,P.Werres,M.D.
Augusta Hospital,Düsseldorf, Germany

Aim of presentation:Carl Langenbusch performed in 1882 the first cholecystectomy,Erich Mühe in 1985 the first cholecystectomy laparoscopically.The principle of both operations is the same,their performance quite different.The intention of this presentation is to offer a help to young colleagues to exercise these two operations.

Methods: Step by step we show the both operations,compare them and show the difference between them.First the laparotomic cholecystectomy in its individual phases,then the laparoscopic way of cholecystectomy.

Conclusions:Advantages of the endoscopic surgery: 1. minor traumatisation of the abdominal wall and of the peritoneum,2.very good inspection of the whole peritoneal cavity,3. minor postoperative pain,4.rapid recovery of the patients.

Advantages of the laparotomic surgery: 1.in cases of complicated gallbladder diseases it is the method of choice,2. the same in malignant tumors of the gallbladder, 3. it permits to remove every ill gallbladder by an wide access to the ill tissues and direct contact.

Only a systematic preparation can lead to operations with few risk and full success.

### V088

### CHOLEDOCHAL CYST AND ACUTE PANCREATITIS.

M.A. Secchi

Surgical Division "B".Hospital Italiano.Rosario.Argentina

Reported Clinical Case. Videofilm Pal-N, 12 minutes.

The patient: Female patient. 17 years old. Admission: moderate acute pancreatitis (AP).

Procedures: Ultrasound and CT Scan: Gallbladder lithiasis and choledochal cyst lithiasis. M.R.I. with cholangiography: Choledochal fusiform cyst (TypeI- Alonso-Lej classification). ERCP was not performed because of cyst infection risk.

The surgery was performed at day 14. The pancreas with edema and Type I choledochal cyst was found. The intra-operative distal cholangiography was normal (no papillary obstruction).

A cyst excision and Roux-en-Y hepaticojejunostomy were performed.

Results: there occured a hemorragic ascitis, treated with peritoneal drainage with favourable evolution. Recurrent pancreatitis was not detected. Postoperative hospitalization time was 10 days.

Discusion: Choledochal cyst is frequently associated with AP (10-20%) but it is a rare cause of AP in our series (0.35%:1/266). A precise diagnosis was made through IV cholangiopancreatography by M.R.I. This is safer than ERCP because there no risk of cyst infection.

Radical treatment is the most appropiate one to prevent cancer and recurrent AP.

### V089

### RESTAURATION OF MAIN BILE DUCT WITH POLYTETRAFLUORETHILENE TUBULAR GRAFT-experimental study

M.Stojanovic, M.Jeremic, P.Stojanovic, M.Stojiljkovic, V.Zivkovic, V.Katic, V.Djordjevic, M.Pesic, Z.Cvetkovic
SURGICAL CLINIC, CLINICAL CENTER NIS, YUGOSLAVIA

The aim of this study is to analyze feasibility and succes of restauration of hepaticocholedochus-HCH with synthetic tubular graft. In fifteen dogs,the authors performed a resection of the supraduodenal HCH, 1 cm in length and arising defect was bridged over polytetrafluorethilene tubular graft a 4 mm. Fifth months living rate was 45% and most ussualy complications were leakages of the anastomoses(33%), and early rejection of the graft.(11%). The animals were followed-up 150 days with the control of holestasis and hepatocellular necrosis parameters and amylase level, which values were increased in the first postoperative days, with normalisation during the forth week. Histological examinations performed every 7 days showed adequate epitelisation in 22%, but intensive fibrous reactions in some cases. Intraoperative transpapillary cholangiography performed on 5 dogs after 150 days showed absolute functionality of the graft in 22%, high-percent stenosis in 11% and total obstruction in 11%.Inspite of many theoretical advantages of restaurative surgery of HCH ,using synthetic tube, basic problems remains great percent of anastomotis leakage and adequate epithelisaton of the PTFE. Protection of the grafts and anastomoses using intraluminaly prothesis and omentoplasty probabily decrease rate of fibrous stenosis,what is our present experimental work in course.

### V090

### COLOR DOPPLER SONOGRAPHY IN PATIENTS WITH ACUTE CHOLECYSTITIS AND DECISION: OPEN OR LAPAROSCOPIC CHOLECYSTECTOMY.

Szymczuk J., Ladny J.R., Polaków J., Krejza J.,Rog M., Puchalski Z.
Department of General Surgery, Medical Univeristy of Bialystok, Poland

Laparoscopic cholecystectomy (LC) has become the standard operation for symptomatic gallbladder disease. Acute cholecystitis remains a challenging problem for most surgeons, and the reported rate of conversions to open surgery is still high.

The aim of this study was to analyze the color Doppler imaging features and clinical importance of inflamed pericholecystic fat.

*MATERIAL AND METHODS:* Forty five patients with surgically and histologically proved right upper quadrant inflammatory lesions in the gallbladder or the pericholecystic space underwent color Doppler sonography (CDS). Findings in the pericholecystic space were correlated with those at computed tomography in four patients and with surgical findings in 45 patients.

*RESULTS:* CDS performed in 15 (33%) of the 45 patients demonstrated echogenic pericholecystic masses greater than 1 cm in diameter that contained internal vascularity. CT in four patients and surgical findings in all 15 patients demonstrated inflamed fat adherent to the gallbladder. All patients were submitted to early LC, whereas 15 patients were managed conservatively and underwent elective laparoscopic operations. Conversions rate was 3/15 ( 20%) patients after elective operation and 1/30 (3.3%) after early operations.

*CONCLUSION:* Identification with CDS of inflamed pericholecystic fat may provide preoperative information that could be pertinent in the decision to perform open or laparoscopic cholecystectomy in patients with acute cholecystitis.

### V091

### EXTENDED RIGHT LOBECTOMY WITH CAUDAL AND PORTAL COMBINED RESECTION AFTER EMBOLIZATION OF THE RIGHT PORTAL VEIN FOR CANCER OF THE PROXIMAL BILE DUCT

S. Tashiro, S. Yogita, D. Wada, M. Harada, T.Matsumura, Y. Fukuda, H. MIYAKE, M.Ishikawa, M. Oka

1st Dept. of Surgery, University of Tokushima, Tokushima 770, Japan

Carcinoma arising at the confluence of the right and left hepatic duct is problematic because of the difficulty of resection and reconstruction. Resection is the best aviable treatment, but long-term results of resection remain poor because of frequent intraductal and periductal tumor spread. From analyzing of 39 resected cases, it became obvious that residual cancer cells of the hepatic duct after resection was most important prognostic factor. Therefore radical resection should be performed, including major hepatic resection with caudal resection and occasionally even combined vascular resection. However major hepatic resection such as extended right lobectomy or right trisegmentectomy is likely to result in postoperative hepatic failure. So when extended right lobectomy or right trisegmentectomy undergo, we perform embolization of the right portal vein to promote regeneration of the left lobe preoperatively.

I would like to present with video the procedure , extended right lobectomy with caudal and portal combined resection, that was performed after embolization of the right portal vein for cancer of the proximal bile duct in a 71-year-old female patient.

